# Fluorescent Probes for Nucleic Acid Visualization in Fixed and Live Cells

**DOI:** 10.3390/molecules181215357

**Published:** 2013-12-11

**Authors:** Alexandre S. Boutorine, Darya S. Novopashina, Olga A. Krasheninina, Karine Nozeret, Alya G. Venyaminova

**Affiliations:** 1 Muséum National d’Histoire Naturelle, CNRS, UMR 7196, INSERM, U565, 57 rue Cuvier, B.P. 26, Paris Cedex 05, F-75231, France; E-Mail: karine.nozeret@mnhn.fr; 2 Institute of Chemical Biology and Fundamental Medicine, Siberian Division of Russian Academy of Sciences, Lavrentyev Ave., 8, Novosibirsk 630090, Russia; E-Mails: danov@niboch.nsc.ru (D.S.N.); okrasheninina@gmail.com (O.A.K.); ven@niboch.nsc.ru (A.G.V.); 3 Department of Natural Sciences, Novosibirsk State University, Pirogova Str., 2, Novosibirsk 630090, Russia

**Keywords:** fluorescent probes, living cells, RNA, DNA, imaging, triplex-forming oligonucleotides, minor groove binders, fluorophores, molecular beacons, binary probes

## Abstract

This review analyses the literature concerning non-fluorescent and fluorescent probes for nucleic acid imaging in fixed and living cells from the point of view of their suitability for imaging intracellular native RNA and DNA. Attention is mainly paid to fluorescent probes for fluorescence microscopy imaging. Requirements for the target-binding part and the fluorophore making up the probe are formulated. In the case of native double-stranded DNA, structure-specific and sequence-specific probes are discussed. Among the latest, three classes of dsDNA-targeting molecules are described: (i) sequence-specific peptides and proteins; (ii) triplex-forming oligonucleotides and (iii) polyamide oligo(N-methylpyrrole/N-methylimidazole) minor groove binders. Polyamides seem to be the most promising targeting agents for fluorescent probe design, however, some technical problems remain to be solved, such as the relatively low sequence specificity and the high background fluorescence inside the cells. Several examples of fluorescent probe applications for DNA imaging in fixed and living cells are cited. In the case of intracellular RNA, only modified oligonucleotides can provide such sequence-specific imaging. Several approaches for designing fluorescent probes are considered: linear fluorescent probes based on modified oligonucleotide analogs, molecular beacons, binary fluorescent probes and template-directed reactions with fluorescence probe formation, FRET donor-acceptor pairs, pyrene excimers, aptamers and others. The suitability of all these methods for living cell applications is discussed.

## 1. Introduction

The mechanisms of cell development, reproduction, differentiation, functioning and evolution, as well as those of gene expression in living organisms are important subjects of modern molecular and cellular biology researches. Direct imaging of events in individual cells [1,2] and even in living organisms [3] is one of the most widely used approaches for these studies. In general, any experimental intervention into intracellular processes leads to their modifications and distortion, thus the results may not adequately reflect the real situation in living organisms. In experimental research it is impossible to avoid completely such interventions, therefore such distortions have to be minimized. In this way, an imaging approach is less invasive and more suitable for observing individual events in cells compared to traditional biochemical methods that give averaged results for a pool of cells after specific experimental treatment that can affect native intracellular processes.

Storage, transfer, exchange and expression of genetic information in all organisms are realized through nucleic acids (DNA and RNA). At present, different classes of nucleic acids are being discovered and described, especially regarding RNA. A great volume of information beginning from primary structures up to fine regulation mechanisms in complex biological systems has accumulated in different publications and databases. Moreover, new high throughput sequencing methods [4] permit one to obtain huge volumes of data concerning DNA and RNA primary structures and to reveal coding and non-coding regions, epigenetic modifications, *etc*. Finally these data also provide great progress in the understanding of genome organization in living organisms [5].

Direct visual observation of nucleic acids *in vivo* requires new specific and sensitive probes for studies of DNA and RNA structure and their interactions with other cell components, their stability, their mobility and dynamics, and finally their functions in living cells. Construction of new instruments for rapid and reliable visual detection of genetic disorders, mutations and mobile genetic elements is also an important practical issue of these researches. This will open the way for new diagnostics of genetic diseases, cancers or viral and bacterial infections, as well as for the artificial regulation of genetic expression in living cells by using specific probe conjugates with oligonucleotides, peptides, biologically active substances, chemical or photochemical reactive agents and toxic drugs.

The direct observation of native double-stranded DNA in living cells is especially interesting. Limited information is available about the real structure of DNA in chromosomes and the fine mechanisms of DNA movement and rearrangement during the cell cycle. Labeling of specific DNA regions, such as centromeres, telomeres or other repeated sequences, by stable tightly bound probes that do not disturb drastically their biological properties will allow to observe them in dynamics in real time and to get ideas about their functional roles. With the improvement of method sensitivity, one can hope not only to detect separate genes, transposons or non-coding regions in real time and environment, but also to observe their availability in dynamics and to make conclusions about their interactions, their reciprocal movement and finally about mode of their functioning.

A large part of the publications concerning live cell imaging deals with the localization and visualization of intracellular structures such as cellular organelles, chromosomes, proteins and small molecules, as well as total DNA and RNA. The existing methods are either label-free or involve labeled probes. Raman confocal microscopy [6,7] is an example of label-free imaging in living cells. Using immunofluorescence signals as references, Klein *et al*. [6] managed to identify cell components such as nucleus, endoplasmic reticulum, Golgi apparatus and mitochondria by Raman spectroscopy. Construction of a Raman microscope equipped with both a slit-scanning excitation and detection system and a laser beam steering and tracking device has been described and used for the detection of small molecule tags and for studying the dynamic properties of cells, such as cellular transport pathways and DNA synthesis [7]. 

An example of non-fluorescent imaging with labeled biologically active molecules is the positron emission tomography (PET) technique, which is based on the labeling by radionuclides–positron emitters [8]. The isotope ^18^F is often applied for substrate labeling *in vitro* and *in vivo*. The use of ^18^F-glucose allows labeling of biological molecules *in situ*. In case of nucleic acids, chemical labeling by positron-emitting isotope reagents is used [8,9].

Fluorescent labeling and fluorescence microscopy are the most common methods for live cell imaging. Great improvements have been achieved in image resolution (see review [10]). Discovery of Green Fluorescent Protein (GFP, for which the 2008 Nobel Prize in Chemistry was awarded to Shimomura Chalfie, and Tsien) and the improvement of the protein properties by multiple mutations and development of a multicolor imaging with fluorescent proteins [11,12,13,14] have provided significant advances in this area. It has been shown that gene-engineering fusion of functional proteins with GFP and other fluorescent proteins does not destroy biological and fluorescent properties of both fused components. It has also opened a new way for DNA and RNA imaging by fluorescence microscopy. 

Modern microscopes are very sensitive and sophisticated instruments. However, their sensitivity is not high enough to detect a signal from one fluorophore molecule bound to a unique DNA or RNA sequence. Thus the target sequence must be relatively abundant. In case of RNA, it is a question of a number of transcribed copies. For genomic DNA, repeated DNA sequences are the most convenient objects for probing. In a majority of studies, these repeats that are localized in specific loci of chromosomes, such as centromeres or telomeres, have been used as the targets. Local concentration of the desired sequence is quite high and can be detected with the fluorescent probes. One class of repeats is found in centromere and pericentromere regions of chromosomes [15]. Their functions are not yet completely understood. They participate in sister chromatid segregation and cohesion and in the coordination of chromosome movement during mitosis, but there are also evidences that they could have many other unknown functions. The centromeres have two types of repeats: major and minor satellites. These repeated sequences cover several millions of base pairs. The major satellites are 10–20 times larger in number [15]. Telomeres are localized at the ends of chromosomes; they play an important role in the cellular life cycle. Their length is shortened with cell ageing. The maintenance of telomere length leads to the cell immortalization, so the size of telomere regions is directly linked to the oncogenesis [16,17,18].

The following requirements must be taken into account when designing fluorescent probes in view of their use for nucleic acid imaging in living cells:

- high affinity and specificity for the target nucleic acid sequence,
- good cellular penetration and ability to reach its nucleic acid target in cells,
- stability of probes under physiological conditions,
- minimal cytotoxicity,
- minimal distortion of cellular functions,
- simple detection in living cells after excitation with non-harmful visible light,
- modulation of fluorescence spectra upon interaction with a target,
- high signal to background ratio.

This review deals with the fluorescent probes used in fixed and living cells that are adapted or could potentially be adapted for direct dynamic observation of native DNA and RNA during cell cycle using fluorescence microscopy. It covers the principles of probe design for this purpose as well.

## 2. Probes for Double-Stranded DNA Imaging by Fluorescence

### 2.1. DNA Imaging in Fixed Cells and Method FISH

Currently, progress in sequence-specific DNA imaging by fluorescence microscopy has been achieved in a large part by employing the fluorescent hybridization *in situ* (FISH) method [19,20,21,22]. This technique is based on a chemical fixation of cells and denaturation of DNA, followed by hybridization of denatured single-stranded DNA inside the cells with fluorescent probes (labeled oligonucleotides or long DNA fragments). It provides a unique opportunity to study nucleic acids directly in the context of their nuclear environment. Labeled PCR fragments and synthetic oligonucleotides, as well as modified analogues, such as PNA [23,24,25] or LNA [26,27,28,29] can be used as hybridization probes. An increased sensitivity can be achieved by using combinatorial mixtures of labeled oligonucleotides targeted to one gene (COMBO-FISH) [30,31,32]. The development of this technique significantly contributes to improvement of our understanding of the cell nuclear organization. However, the FISH method is not compatible with the observations in living cells.

### 2.2. Non-Specific DNA Detection and Staining

**Figure 1:**
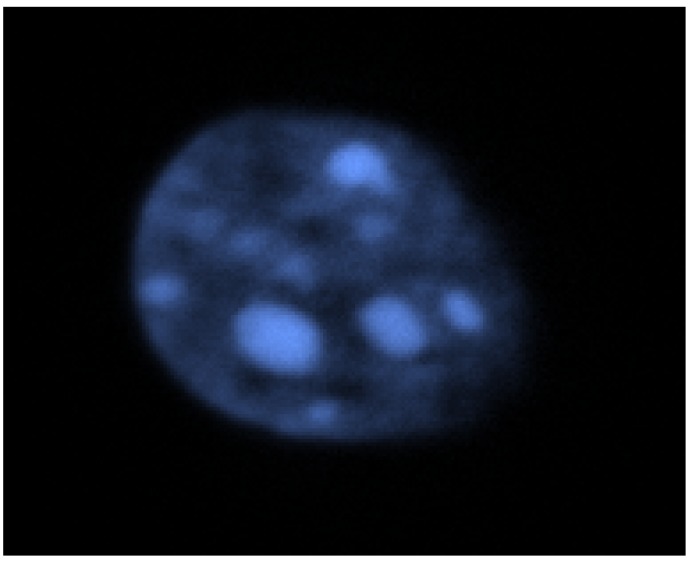
DNA visualization in fixed murine 3T3 cell nucleus using minor groove binder 4',6-diamino-2-phenylindole (DAPI). The image was kindly provided by Dr. C. Escudé (CNRS, UMR 7196, Paris, France).

Non-specific double-stranded DNA detection and visualization can be monitored using intercalating [33,34] or minor groove-binding fluorophores, such as 4',6-diamidino-2-phenylindole (DAPI, Figure 1) [35], Hoechst 33258 and others [34]. Among several studied fluorophores, only Hoechst 33258 and DRAQ5 demonstrate good cell penetration properties and are suitable for live cell DNA staining [34]. 

### 2.3. Sequence-Specific DNA Labeling

**Figure 2:**
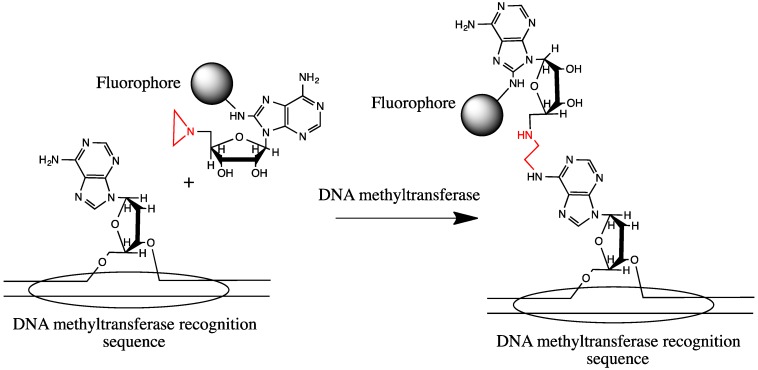
DNA sequence-specific labeling using DNA methyltransferase and fluorescent aziridinyl derivative of adenosine as its substrate instead of S-adenosylmethionine [36,37,38].

The Weinhold group has proposed an interesting method of the sequence specific dsDNA labeling using natural sequence-specific enzymes and synthetic substrates (Figure 2). DNA methyltransferases are enzymes that methylate specific sequences of the target DNA using S-adenosyl methionine as a substrate. In the case of the Weinhold’s approach, instead of S-adenosyl methionine, an artificial fluorescent substrate with the chemically active aziridine group was used. Thus, instead of methyl group, a fluorescent label can be inserted into DNA target sequence [36,37,38]. However, this method is too far from the *in vivo* applications because it requires the presence of methylases of desired specificity in cells, as well as a synthetic chemical substrate that can be cytotoxic.

### 2.4. Fluorescent Antibodies for DNA Imaging

The use of structure-specific antibodies against C5-bromouridine inserted into nascent DNA *in situ* [39], against chromosomal loci [40] or against specific DNA structures [41] allows visualizing *de novo* DNA synthesis or particular DNA structures and complexes (centromeres, telomeres, G-quadruplexes). The antibodies are not really sequence-specific. They are more suitable for fixed and permeabilized cells because of their bad cell penetration. However, this technique is quite powerful. It has allowed, for example, to demonstrate for the first time the existence of G-quadruplexes not only *in vitro*, but in cellular environments [41].

### 2.5. Fused GFP and Other Color Proteins as DNA-Specific Probes

**Figure 3:**
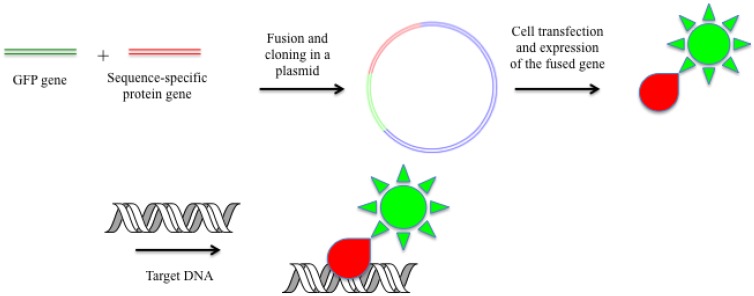
DNA imaging in living cells with fused proteins containing GFP and site-specific or sequence-specific proteins.

Concerning the direct observation of specific DNA sequences in living cells, much less publications and achievements can be cited as compared to the FISH method. Approaches for DNA visualization are based mainly on the use of artificial gene constructs engineered to express different specific DNA-binding peptides and proteins fused with fluorescent proteins (GFP and other “colored proteins”) directly in cells (Figure 3). In this area, a very impressive work has been done by Sugimoto *et al*. [42]. They managed to express in living cells five different DNA-binding proteins participating in the chromosome segregation (CENP-A, centromere-specific histone, histone H3, importin-α and α-tubulin) fused with fluorescent proteins of various colors. The result of this work was a direct observation by multicolor fluorescent microscopy of the chromosome segregation dynamics upon mitosis in modified human cancer cells. Engineered gene constructs expressing DNA-binding proteins targeted to specific bacterial DNA regions and fused with GFP have also been used for studying of bacterial chromosomes [43].

As previously mentioned, telomeres are attractive objects for direct intracellular observations. As telomere-specific probes, proteins of the telomere complex, such as TRF1 and TRF2, fused with fluorescent proteins are used [44,45]. This approach involves the development of gene constructs and modified transfected cell strains. It is not simple and easy in application. In addition, target DNA after interaction is not in a native state, but in a complex with voluminous proteins. This is against the principle of “minimal intervention”.

### 2.6. Hybridization with Oligonucleotide Analogs in Living Cells

Sequence-specific fluorescence imaging of native DNA in living cells is not an easy task. In the native state, genome DNA is a double-stranded molecule and a part of the complex chromatin structure. Thus it is not accessible for hybridization with complementary oligonucleotide probes. The DNA should be either denatured or targeted by special probes recognizing specifically DNA sequences in a double-stranded state without denaturation. Otherwise, this approach relies on existence of locally depleted or single-stranded DNA regions (as in telomeres that have single-stranded DNA at their 3'-ends). Local DNA unwinding can also proceed during some dynamic rearrangements and processes such as transcription, recombination or repair. 

The use of fluorescent peptide nucleic acids (PNA) for telomere probing has been described [46]. PNA can also interact with DNA via triple helix formation or strand displacement generating duplex-loop structure that disturbs the native DNA structure. In addition, PNA has a serious drawback: it does not penetrate into living cells and requires using of microinjection, permeabilization by pore-forming toxins (as streptolysin-O) or other invasive cell delivery methods [47].

Several examples of modified oligonucleotides probes suitable for detection of both ssDNA and RNA will be discussed below in the Section 4. Triple helix formation of linear oligonucleotides and their analogues with dsDNA is reviewed in Section 2.8.

### 2.7. Sequence-Specific Proteins Recognizing Double-Stranded DNA (Zinc Fingers, TALE)

**Figure 4:**
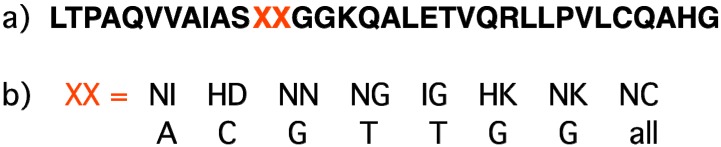
(**a**) Primary structure of the TALE element recognizing one base pair. 12-th and 13-th amino acids (XX in red) determine the element specificity. (**b**) Recognition code of the TALE elements [55].

To date, several classes of natural and synthetic compounds are known to recognize specific sequences of the double-stranded DNA and to bind them [48,49,50,51]. One natural source of DNA-recognizing peptides is the family of proteins called transcription factors. Several polypeptide probes and artificial nucleases have been constructed on the basis of these factors using gene-engineering methods. In the beginning of the 2000s many laboratories were developing “zinc fingers”, peptide structures that originate from the DNA-binding domains of eukaryotic transcription factors (“zinc proteins”) containing zinc ions linked to helical peptide structure through two histidines and two cysteines or through four cysteines [51,52,53]. Using gene engineering, specific fluorescent probes for live cell imaging in plants (*Arabidopsis*) and centromere repeats in mouse were constructed from a specific polydactyl zinc finger fragments fused with the green fluorescent protein (GFP) [54]. However, currently, zinc fingers are progressively replaced by new sequence-specific peptides called transcription activator-like effectors (TALE). These proteins come from transcription factors of plant bacteria *Xanthomonas spp* that are able to change genetic expression in the host plants [55,56]. Each element of a TALE polypeptide contains 33–35 amino acids. The nature of the 12-th and 13-th amino acids determines the recognition code (Figure 4); one specific combination of the amino acids in these positions corresponds to one base pair. This property of TALEs has been used for the construction of artificial nucleases (TALEN) by fusing TALEs with the cleaving fragment of Fok1 nuclease [57,58,59]. TALEN are able to modify genomic DNA by the induction of directed mutations. The spatial structure of the DNA-TALE complexes has been elucidated by crystallography [56]. This is also an opportunity for construction of fluorescent probes based on the fusion of TALEs with fluorescent proteins. 

However, design of zinc fingers and TALEs is not a simple procedure. Like for structure or region-specific probes, it must include gene-engineering operations, such as plasmid construction and cloning, fusion and insertion of TALE and fluorescent protein genes, verification of fluorescent and functional properties of the resulting proteins and then cell transfection.

### 2.8. Triplex-Forming Oligonucleotides

**Figure 5:**
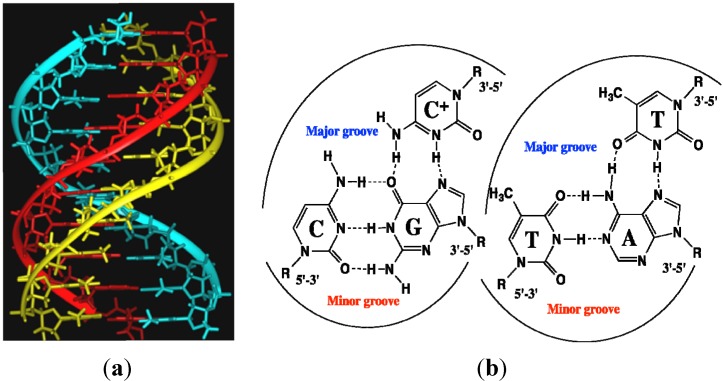
(**a**) DNA triple helix: polypurine strand (red), polypyrimidine strand (cyan) and third polypyrimidine strand (yellow) in a major groove of the duplex. (**b**) Examples of Hoogsteen triplets C:G-C and T:A-T.

Besides natural peptides and their engineered derivatives, only two classes of synthetic molecules are able to recognize and to bind double-stranded DNA sequence-specifically without duplex denaturation. They are triplex-forming oligonucleotides (TFO) [60,61] and N-methylpyrrole/N-methylimidazole polyamide minor groove binders [62,63]. TFO can recognize long (12–30 base pairs) oligopurine tracts and bind to dsDNA major groove via Hoogsteen or reverse Hoogsteen hydrogen bonds (Figure 5). In order to form an additional hydrogen bond, they require slightly acidic pH for cytosine protonation. In physiological conditions, triple helices are often quite unstable [64,65,66,67,68,69]. In addition, the intracellular penetration of the nucleic acids requires the use of transfection agents or mild methods of permeabilization. On the other hand, oligonucleotides are easy to synthesize and they are commercially available. Moreover, their labeling by fluorophores is well developed [70,71].

**Figure 6:**
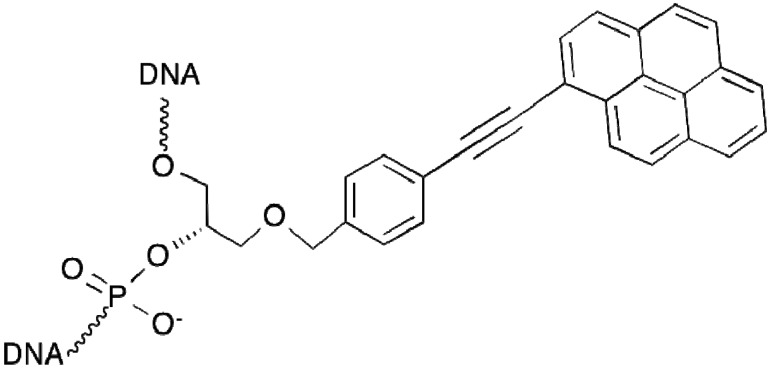
TINA (Twisted Intercalating Nucleic Acid) monomer.

Several years ago a new class of triplex-forming oligonucleotides was synthesized: twisted intercalating nucleic acids (TINA) [72,73,74,75]. Due to presence of the intercalating pyrene residue in a special spatial configuration, these oligonucleotides form stable triplexes with target dsDNA; the antiparallel triplexes being more stable compared to the parallel ones. The presence of several pyrene residues in the third strands prevents G-quadruplex formation and provides implication of G-rich sequences into triple helix, in contrast to “natural” oligonucleotides [75]. In addition, the pyrene moiety is naturally fluorescent (Figure 6).

Fluorescent triplex-forming oligonucleotides have been used for microscopic detection of dsDNA [76,77]. Additionally, TFO have been applied in COMBO-FISH [32] and TISH methods (TISH, triplex *in situ* hybridization) [78]. TISH method has been also adapted to physiological temperatures and pH (C. Escudé, private communication). However, all these works have been done with fixed cells because the background fluorescence of non-bound oligonucleotides is too high, and intensive washing of cells is necessary before imaging to remove their excess and to decrease background fluorescent signal. The forthcoming progress in live cell experiments requires new methods and probes allowing the observation of only target-bound molecules. The main problem of all the native and modified triplex-forming oligonucleotides is their weak intracellular delivery. Development and application of new soft transfection reagents and procedures, as well as mild methods of probe delivery to target sites are needed.

### 2.9. Polyamide N-methylpyrrole—N-methylimidazole Minor Groove Binders

**Figure 7:**
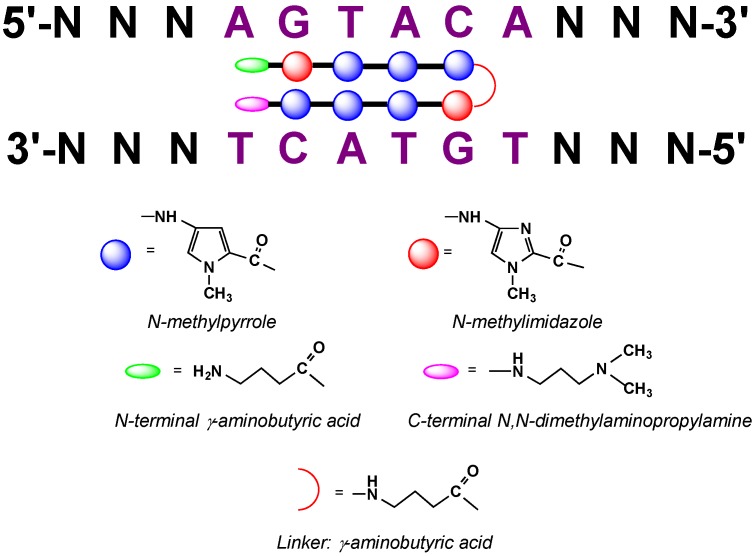
Structure of hairpin N-methylpyrrole/N-methylimidazole polyamides and code of DNA recognition upon their binding to DNA minor groove [79].

Polyamide minor groove binders (MGB) seem to be the most promising candidates for dsDNA sequence-specific intracellular targeting. The classic commonly used polyamides consist of two blocks of N-methylpyrrole (Py)/N-methylimidazole (Im) carboxamides linked by γ-aminobutyric acid [79] or other linkers [80,81,82]. These two blocks form an antiparallel hairpin in which each pair of carboxamide residues is a base pair recognition unit (Figure 7). Polyamide minor groove binders interact with the DNA via hydrogen and van der Waals bonds. The recognition code is quite simple: Py/Py pair recognizes A-T or T-A, Py/Im–G-C, and Im/Py–C-G. Additional pyrrole ring modification or insertion of different heterocyclic aromatic rings allows distinguishing between A-T and T-A pairs [79,83]. Their synthesis is relatively simple [84,85,86]. In contrast to TFO, they are not limited by the particularities of the target sequence, their interaction is not dependent on pH and salt concentrations [87,88,89,90], and they have high affinity for dsDNA but lower specificity because of their shorter length. For a standard hairpin ligand the length of the recognized sequence is no more than 6 base pairs. In order to increase their affinity and specificity for target DNA, different chemical modifications can be introduced. Often the polyamides are conjugated to other gene-specific molecules as TFOs or other MGBs [91,92,93,94]. Good results were obtained by using tandem systems, where two or more hairpin polyamides interacting with neighboring DNA sequences are linked covalently [95,96,97]. These modifications do not only increase the ligand specificity, but also improve their affinity [97]. 

Different functional groups can be inserted into the terminal and central positions of the polyamide molecules for their additional chemical modifications, including fluorophore labeling. Fluorescent MGBs have been used to study their interactions with DNA and to detect DNA *in vitro* and in living cells [98,99,100,101,102,103,104,105,106,107]. For review, see [108].

Research in the last decade has demonstrated the ability of MGB to penetrate into living cells without transfecting agents and to reach their intracellular targets, thus they are good candidates for the application in living systems [81,104,108,109,110,111,112]. It was found that MGB uptake and *in cellulo* distribution, as well as their pharmacokinetics, toxicity and biodistribution in organs *in vivo*, depend on structural peculiarities of polyamides: pyrrole/imidazole contents and their order, nature and size of the linker between two oligopyrrole/oligoimidazole blocks, replacement of N-methylpyrrole residue by ß-alanine, modifications at the polyamide termini, polyamide cyclization, *etc*. [81,105,113,114,115]. 

The ability of fluorescent polyamide tandems with enhanced specificity to label the telomeric repeats was demonstrated by the Laemmli’s laboratory using the FISH method in fixed cells of insects and vertebrates [107]. Another polyamide tandem against human and mouse telomeres was recently synthesized by Sugiyama’s team and successfully applied for labeling of telomeres in mouse MC12 and human HeLa cells [116]. Given the ability of minor groove binders to penetrate into living cells, these works open the way to their use *in cellulo*. Dervan *et al*. have already applied ^18^F-labeled polyamides for positron emission tomography *in vivo* imaging to study their biodistribution in mice [9]. However, the main difficulty of fluorescence microscopy imaging is a very high background of non-bound probe in living cells. While fixed cells could be easily washed from non-bound probes, in living cells other approaches have to be used. May be, it is a reason, why there are not so many publications on the subject. One can cite the work of Hsu and Dervan [104] to determine polyamide concentration in cells, as well as a recent publication by Peng *et al*. [110], who have used a non-specific minor groove binding probe different from sequence-specific polyamides.

## 3. Choice of Approaches and Fluorophores for Live Cell Applications

As it has been already mentioned, an important requirement for living cell observations is a possibility to detect a real signal from target-bound probes on the background fluorescence of non-bound molecules that penetrate into cells. It is a problem for both DNA and RNA *in cellulo* detection. 

In order to circumvent the problems of the high fluorescence background, several approaches have been proposed. The first way is to use single linear probes and fluorophores that change their fluorescence spectra upon interaction with the target DNA, *i.e.*, fluorescence intensity (light-up probes) and/or maximum of emission spectrum (red or blue shift) [77,103,117,118]. In this case, the desired signal will be seen as a bright point or as a point of different color within the fluorescent background. For example, linear perylene-conjugated pyrrole polyamide has been shown to be a sequence-specific light-up probe [103]. It becomes fluorescent in the presence of DNA, especially upon formation of a sequence-specific heterodimer complex with the other linear polyamide in the minor groove of the telomere DNA fragment. Authors have demonstrated that upon interaction with DNA a transfer of energy occurs between the pyrrole moiety and the perylene residue, giving rise to a strong fluorescence emission.

**Figure 8:**
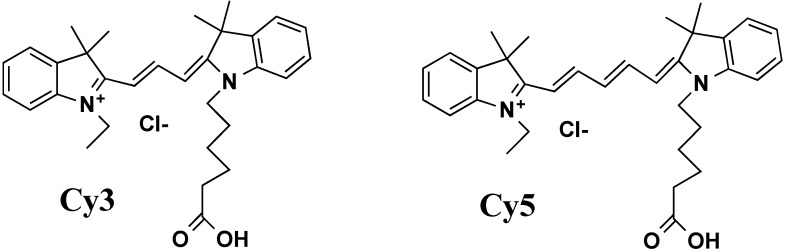
Cyanine fluorophores Cy3 and Cy5.

However, the popular fluorophore fluorescein and also several other widely used fluorescent dyes do not possess the desired properties. According to the literature, the best fluorophores that fulfill these requirements are the cyanine dyes [119,120]. A large variety of these dyes is accessible commercially. Among them, the most widely used are thiazole orange [77,100,117,118], Cy3 and Cy5 [121] (Figure 8). New synthetic polymethine cyanines developed by Yarmoluk’s laboratory are also promising [122,123,124,125,126]. They change both fluorescence intensity and emission spectra upon interaction with nucleic acid targets. 

**Figure 9:**
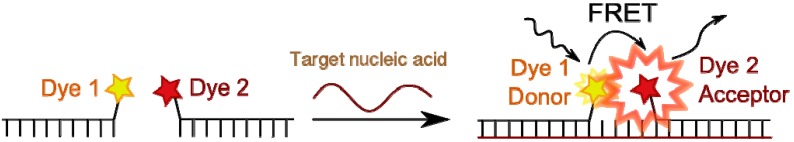
Fluorescence Resonance Energy Transfer (FRET).

The other way is to use fluorescence resonance energy transfer (FRET) [127,128]. When two fluorophores (donor and acceptor) are in the close proximity, excitation of the donor results in transmission of the energy to the acceptor, which emits light with a different spectrum. If the light filter does not transmit the donor background emission, only acceptor fluorescence is seen when the donor is excited (Figure 9). Couples of cyanine dyes, for example Cy3 and Cy5, are able to transfer excitation energy and can be used for FRET experiments [46,129,130].

Pyrene is a molecule that is excited at 340–345 nm, and emits fluorescence at 370–400 nm. However, when two pyrene residues are in close proximity, they form a complex called excimer that emits fluorescence at 480–485 nm [131]. The use of pyrene couples attached to probes is another approach, conceptually similar to FRET. In addition, pyrene excimer fluorescence can increase upon hybridization with nucleic acids, a property that can be exploited for RNA or single-stranded DNA [132,133,134]. The drawback of using pyrene is its requirement for near-ultraviolet excitation light that can be damaging for cells, does not penetrate into tissues and can produce background protein fluorescence.

In general, the use of FRET between two fluorescent molecules is a particular case of so called “binary probe” approach, where two components of the probe directed to the adjacent regions of the target nucleic acid form a complex generating fluorescence signal. For example, such complex is able to bind a specific fluorophore due to formation of its binding site formed by two components, or generate (catalyze) a reaction leading to the appearance of fluorescence in the targeted region (for details, see Section 4.4.2 and Section 4.4.3).

The “binary probes” approach is described in details in several recent reviews [135,136,137] and, as it was indicated, will be discussed in the next section, because, being suitable for dsDNA, this approach is more frequently developed and used for cellular RNA imaging.

**Figure 10:**
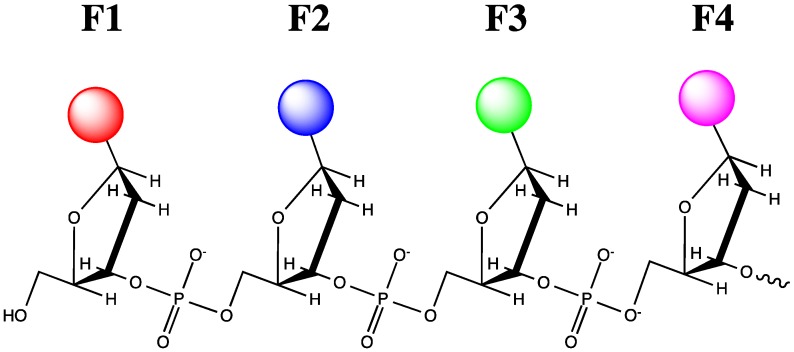
“Oligodeoxyfluorosides” (F1, F2, F3, F4, … —different fluorophore residues).

Concerning multicolor detection, a very interesting series of fluorophores has been developed by Kool’s team. Using DNA synthesizer, they obtained DNA or RNA-like short oligomers (Figure 10) in which different fluorophores are directly attached to a deoxyribose or ribose backbone in place of natural bases [138]. 

These “multichromophores” or “oligodeoxyfluorosides” reveal various fluorescence spectra and emit light of various colors from violet to red after excitation by long-wavelength UV light at 340–380 nm. These oligofluorophores penetrate into cells, accumulate in the cytoplasm and are retained there for a long time. These properties allowed real-time monitoring and tracking of dynamic living biological systems such as zebrafish embryos, invertebrate shrimps *Artemia salina*, ciliate protozoan *Paramecium caudatum* and others [139]. Multicolor fluorescent antibodies have been obtained by conjugation to olidodeoxyfluorosides [140]. Properties and applications of these probes have been described in a recent review [141] and will not be discussed here because these fluorophores have never been used for cellular nucleic acid imaging. It is a little surprising that, to our knowledge, the authors did not yet realize synthesis of sequence-specific probes first with natural nucleic acid targeting sequences and then artificial fluorescent synthons as fluorophores.

## 4. Probes for RNA and Single-Stranded DNA Imaging by Fluorescence

There are many more publications and reviews about live-cell detection and visualization of intracellular RNA than of native dsDNA. Different classes of coding and non-coding RNA accomplish various functions from genetic information transfer to regulation of genetic and epigenetic processes. In the past, the most current subjects of research were messenger, ribosomal and transfer RNAs. However, more recent researches have demonstrated existence of many other different classes of RNA as long non-coding [142], catalytic [143,144], natural antisense [145] and many small non-coding RNAs (nuclear and nucleolar, micro-, Piwi-interacting, small interfering, transacting, small scanning and other RNAs) [136]. They all are non-coding transcripts that play an active role in gene expression and regulation [146,147,148]. There are also non-coding RNAs transcribed from centromere and pericentromere repeats of chromosomes [149,150,151,152,153,154]. Functions of these transcripts are still unknown (for review, see [155,156]). Some of them were demonstrated to affect the interaction of centromeres with chromatin proteins resulting in the distortions of sister chromatid cohesion [149]. Studies of the non-coding transcripts by direct observation is a very intriguing task that can reveal their biological role and functions. It must be noted that a part of probes potentially suitable for single-stranded RNA imaging were developed using the ssDNA as a target. These probes will be also discussed in this section.

### 4.1. Fused Fluorescent Proteins

**Figure 11:**
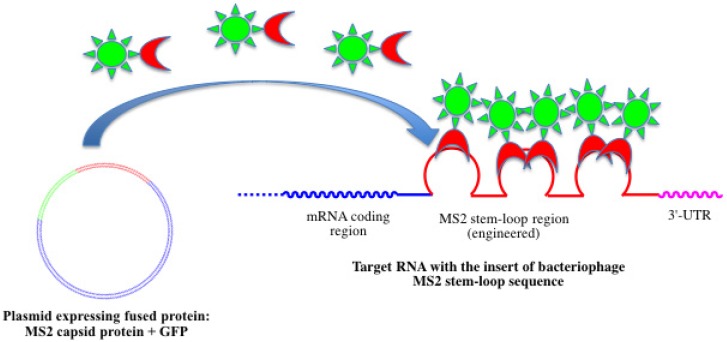
Bacteriophage MS2 genetic encoding system for RNA detection using genetically modified RNA and GFP fused with MS2 capsid protein.

GFP and related fluorescent proteins were adapted for detection and visualization of RNA *in vivo* [136,157,158] (Figure 11). 

Two engineered plasmids are expressed in the living cells. The first one codes for the green fluorescent protein fused with the RNA-binding protein from the MS2 bacteriophage. The second one codes for the reporter RNA that contains in its 3'-untranslated region several copies of a unique hairpin fragment from the MS2 genome that interacts with the MS2 RNA-binding protein. Nuclear localization signal is also inserted into the protein tag. Binding of the fluorescent tag to the reporter RNA can generate a strong fluorescent signal [159,160]. Modified target RNA gene can be integrated into the cell genome by a homologous recombination with PCR-amplified products [161]. This approach has been used to design several genetically encoded systems for the direct observation of intracellular RNA dynamics [157]. However, this method is based on artificial gene constructions and not on natural cell components. It depends on plasmid transfection and intracellular expression of the tags and reporter RNA. In addition, high background fluorescence is observed in this case.

### 4.2. Linear Fluorescent Oligonucleotide Probes

In the case of single-stranded RNA, independently of its structure and functions, only synthetic complementary oligonucleotides or nucleic acid fragments are able to provide efficient sequence-specific recognition. The probe binding has to compete with secondary and tertiary RNA structures and binding of multiple RNA-binding proteins. To date, a large experience has been accumulated in the use of different natural and modified oligonucleotide probes in living cells. We address the reader to several recent reviews in which all the achievements and the problems of the methods are thoroughly described [1,162,163,164]. Here we just briefly list the methods used for RNA intracellular imaging.

**Figure 12:**
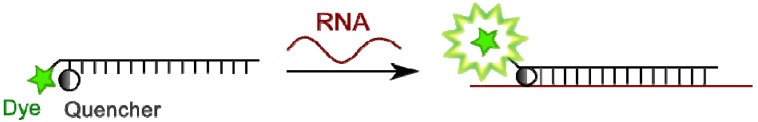
Linear complementary fluorescent probe with fluorophore and quencher at the 5'-terminus. When the quencher intercalates into duplex formed upon hybridization, the fluorescence appears [165].

As in the case of DNA, linear fluorescently labeled oligonucleotide probes are not good enough because of their bad cellular uptake and high background fluorescence, except in cases where the fluorescence is strongly modulated upon the probe-RNA interaction. One interesting example was demonstrated *in vitro* with an oligonucleotide containing both acridine and fluorescein at the same 5'-terminus of the probe [165] (Figure 12). 

When this oligonucleotide is alone, the acridine residue quenches fluorophore fluorescence. In complex with the complementary RNA, acridine intercalates into the duplex formed, and fluorescence is not quenched any more. However, this approach has not been implemented in living cells. In contrast, linear bis-pyrenyl conjugates with pyrene residues attached to 2'-hydroxyls of two adjacent nucleotides in oligo(2'-O-methylribonucleotides) containing phosphorothioate bonds were used for visualization of c-fos mRNA *in cellulo* [166]. An enhanced fluorescence signal was observed due to the property of bis-pyrenyl conjugate to increase its excimer emission upon hybridization with complementary RNA [132,133,134].

Several groups have designed molecular probes with non-natural fluorescent nucleic base analogues and introduced them instead of natural bases into oligonucleotide probes, either into the same strand [167,168] or into two complementary strands of a duplex [169,170,171] at a distance permitting to observe FRET. Hybridization of double-labeled single strands with complementary nucleic acids changes excitonic interaction between two fluorophores, so the color of the probe changes [167]. Wagenknecht’s team has synthesized duplexes with thiazole orange (TO) base surrogate in one strand and thiazole red (TR) base surrogate in the other one in a close proximity. In a double-stranded state, a red light is emitted upon thiazole orange excitation due to the energy transfer from TO to TR. When the duplex dissociates or a strand displacement by a target nucleic acid occurs, no FRET is possible, and light excitation leads to green color emission of TO donor. The difference between two emission wavelength maximums is 140 nm. Using this technique, the authors could monitor penetration of the probes into living cells and their processing both for DNA [169,170] and siRNA [171]. They call these probes DNA or RNA “traffic lights”. However, it is not clear enough how the authors distinguish between probe dissociation/interaction and degradation.

Asanuma *et al*. introduced into probes one or several perylene nucleobase analogues as acceptors and one or several pyrene analogues as donors with D-threoninols as a scaffolds. All the artificial bases were separated by one or several natural ones in order to avoid excimer fluorescence and to observe only FRET [172]. These probes demonstrate a very high Stokes shift and their fluorescence spectra depend on the number of introduced donors and acceptors, on the distances between them and on the hybridization state of the probes [173]. This is an interesting development with high potential; however, it was tested on DNA *in vitro* and never applied to living cells*.*

Okamoto *et al*. modified thymine nucleobase in oligonucleotide probes by attaching two thiazole orange residues to its 5-position. They have obtained a switch on/off probes that are quenched in a single-stranded state due to intramolecular excitonic interaction and develop a strong fluorescence upon hybridization with complementary DNA or RNA [174]. Use of nuclease-resistant oligo(2'-O-methylribonucleotides) possessing this modification permitted them to realize a long real-time monitoring of mRNA in living HeLa cells during all the cell life cycle [175].

Seitz *et al*. introduced into central position of PNA sequence an artificial monomer, which contained fluorophore thiazole orange instead of natural nucleobase [176,177]. The authors called these probes “forced intercalation probes”. Upon hybridization with a complementary sequence, the base surrogate intercalates between base pairs of the duplex with the increase of its fluorescence signal up to 26 times. Moreover, differences in fluorescence enhancement allow distinguishing between completely matched and mismatched duplexes. Using these probes, the authors managed imaging of the neuraminidase (NA) mRNA in permeabilized living cells infected by H1N1 influenza virus [178]. Recently they synthesized two different probes for two mRNAs, NA and matrix protein 1 (M1), labeled by two cyanine dyes, TO (green fluorescence) and BO (red fluorescence), respectively. The probes were applied for simultaneous multicolor monitoring of the temporal and spatial progression of synthesis and dynamics for these two mRNA species in infected living cells [179].

### 4.3. Molecular Beacons

**Figure 13:**
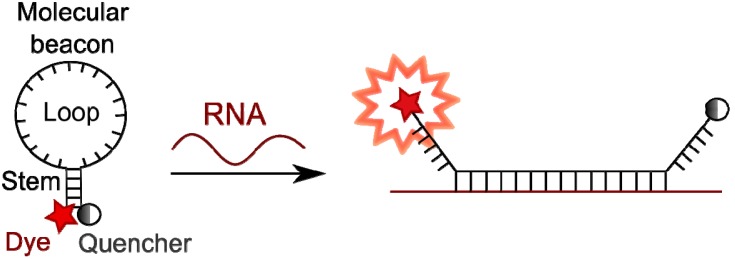
Mode of functioning of molecular beacons.

Molecular beacons are much better adapted for intracellular microscopy observations [180,181,182]. They are stem-loop hairpin oligonucleotides that contain a fluorophore at one terminus and a quencher at the other one. The loop of the hairpin is complementary to the target RNA region, while the stem-forming ends do not interact with it (or only one of two ends interacts) [183,184,185]. In a stem-loop conformation fluorophore and quencher are close to each other and all the fluorescence is quenched. Upon hybridization and formation of a duplex with the target, the hairpin structure opens and the distance between fluorophore and quencher increases and becomes too large for energy transfer. There is no quenching anymore, thus fluorescence is observed (Figure 13). 

The fluorescence intensity can increase up to 200-fold [180], providing a much better signal to background ratio. Competition between hairpin and probe-target duplex formation allows for a better target discrimination compared to linear probes, and hence better sensitivity for single mismatch. Examples of molecular beacon systems for living cell applications are described in several publications [186,187,188,189]. In the paper of Molenaar *et al*. [189], molecular beacons based on oligo(2'-O-methylribonucleotides) were applied for detection of different RNA types (such as ribosomal, small nuclear and specific messenger RNAs) in living cells. The authors have observed intracellular transcription and translation during several days without distortion of the cell life cycle.

For *in vitro* studies, a method of monitoring of cellular enzymatic activity with molecular beacons has been developed. This method is based on the fact that the target sequence (complementary to the beacon’s loop) is able to open the molecular beacon’s hairpin only when this sequence is continuous. When it is cleaved, complementary complex dissociates and the molecular beacon restores its hairpin structure with the fluorescence quenching. Thus, endonuclease cleavage of the target leads to fluorescence quenching, in contrast, target ligation from fragments leads to appearance or enhancement of the signal. Enzymes, which affect the structure of the ends in the cleaved site (polymerases, phosphatases, kinases) can block or, in contrary, restore ligation of the fragments thus “turning off” or “turning on” the molecular beacon’s fluorescence. In this way, the authors managed to monitor in real time the activities of ligases [190,191], endonucleases [192], phosphatases [193,194], DNA polymerases [195], methylases [196] and kinases [197], and also to monitor ATP in biological media [198].

Another class of molecular beacons is represented by quencher-free probes. Molecular beacons with two pyrene moieties at different termini [199,200] or at the same terminus [201] are “excimer-monomer switch-type probes”. They emit excimer fluorescence in a hairpin form, when two conjugated pyrenes are close one to another, but upon hybridization the monomer fluorescence prevails, even when two pyrenes are attached to the same terminus [201]. Further development of molecular beacons is based on the multicolor probes [202,203] and on the new design of probe architecture [193,199,204,205]. In multicolor probes [202], two different fluorophores are attached, one to a 3'-end and one to a 5'-end. In hairpin conformation, there is mutual quenching of both fluorophores (in this paper, they are 1,8-dialkynylpyrene and perylenediimide). In an opened form, upon hybridization with a target, both fluorophores become fluorescent, emitting light of different colors. Tricolor system, in addition to two conjugated fluorophores, includes also an intercalating fluorophore TOTO-3 that enhances its fluorescence upon formation of the duplex between the beacon and its target [203]. Two-color quencher-free molecular beacons were also synthesized by substitution of two nucleobases in 5'- and 3'-parts of the hairpin stem by couple of fluorophores pyrene and Nile Red [206]. Being red in a hairpin form, upon hybridization this probe changes its color to white and then to blue with the shift of maximal emission wavelength up to 225 nm. 

A special case of quencher-free molecular beacons is a pH-sensitive probe. Use of pH-dependent probes has been already described for selective imaging of cancer cells with fluorescent cancer cell-targeted antibodies [207]. In the case of molecular beacon, a pH-sensitive fluorophore 7-hydroxycoumarin was inserted as a nucleobase analogue into the hairpin stem [208]. The pKa of 7-hydroxycoumarin depends on the microenvironment: in a single strand it has pKa 8.8, whereas being intercalated it changes its pKa to 10. Thus, in a double-stranded hairpin stem its fluorescence is quenched. In a complex with complementary strand the stem dissociates, 7-hydroxycoumarin deprotonates and upon excitation emits a fluorescent signal. It must be noted that the experiments have been done on DNA target *in vitro*, but the principle seems to be suitable for RNA.

**Figure 14:**
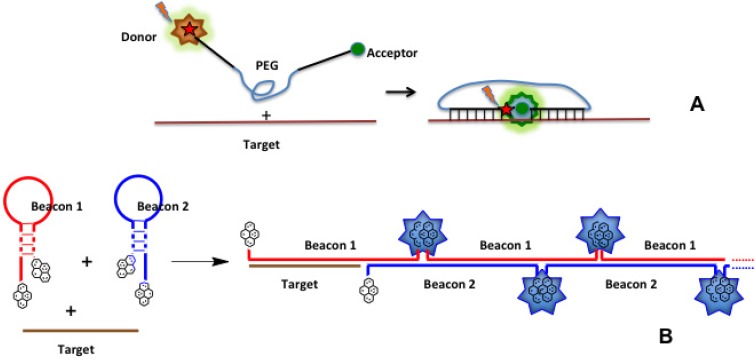
New types of molecular beacons. (**A**) hybrid molecular probe with two antiparallel oligonucleotide strands linked by a long poly(ethyleneglycol) chain [204]. Oligonucleotides complementary to adjacent target sequences are labeled by donor and acceptor fluorophores, respectively. Upon hybridization the FRET effect is observed. (**B**) Hybridization chain reaction [199]. Two molecular beacons are partially complementary and bear two pyrene moieties separated in space. Upon addition of a target, hybridization chain reaction is initiated; two pyrenes from adjacent probes appear in a close proximity and emit excimer fluorescence, which is amplified due to formation of several excimers on one target molecule.

Examples of new design of molecular beacons are shown on Figure 14 (for explanations, see the figure legend). These probes have advantages of better specificity, selectivity and sensitivity (especially the second construct due to signal amplification), modulation of fluorescence spectra upon hybridization, and low level of false positive signals (see below). The last technique in combination with aptamers selected against cell surface proteins allowed to construct fluorescent DNA nanodevices on the target living cell surface [205]. However, it is hardly suitable for detection of intracellular RNA.

The large problem of molecular beacons is the detection of false-positive signals, especially in native biological conditions. There are several reasons for it: (i) degradation of nucleic acid with the release of free or shortened oligonucleotide-bound fluorophores; (ii) unwinding of nucleic acid hairpin by SSB (single strand DNA-binding proteins that open the hairpin and stabilize the single-stranded form by binding); (iii) thermodynamic fluctuations; (iv) sticky end pairing between hybridized molecules [204,209]. In order to stabilize molecular beacons, modified nucleic acids such as peptide nucleic acids (PNA) [210], locked nucleic acids (LNA) and their enantiomer forms [211,212,213,214] or 2'-O-methyl-modified RNA [215] are used (see Section 4.5). Heating above the denaturation temperature in order to denature the SSB protein and use of carbon nanotubes to absorb partially degraded probes due to high affinity of ssDNA to nanotubes is suitable only for *in vitro* applications [209]. As it was mentioned already, new original design of molecular beacons and use of multicolor probes also help to a great extent to solve this problem. 

**Figure 15:**
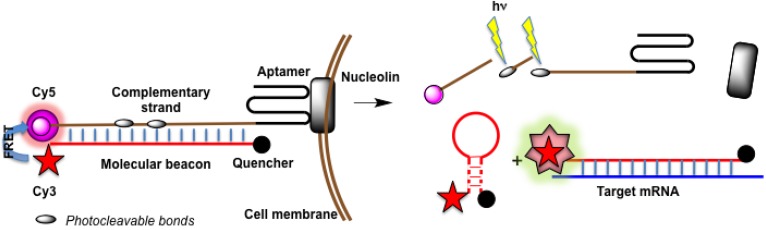
A targeted, self-delivered and photocontrolled aptamer-based molecular beacon [216].

In a very recent publication, Tan’s team proposed a new targeted, self-delivered and photocontrolled molecular beacon for mRNA detection in living cells [216]. This construct (Figure 15) is quite complex, but it works in living MCF-7 cells detecting manganese superoxide dismutase mRNA. The construct consists of two components: a hairpin molecular beacon itself (labeled by fluorophore Cy3 and quencher BHQ2) and a carrier probe for its intracellular delivery. The carrier probe is a DNA fragment containing a sequence complementary to the molecular beacon fused to nucleolin-recognizing internalizing aptamer AS1411. In addition, the complementary sequence has Cy5 fluorophore (to quench Cy3 fluorescence of the molecular beacon) and two photolabile internucleotide bonds that can be cleaved by UV irradiation. On the first step, the whole duplex “molecular beacon–carrier” is formed. Due to the effect FRET, only Cy5 fluorescence is seen in this case. The internalizing aptamer interacts with nucleolin, which transports the duplex inside the cell. After UV irradiation, the photolabile bonds are cleaved, the complementary strand becomes fragmented and dissociates from the duplex. Free molecular beacon either folds into a hairpin conformation with the quenching of Cy3 emission, or forms a complementary complex with the target RNA. In the latter case, a fluorescence signal of Cy3 fluorophore is observed. 

### 4.4. Binary Probes

#### 4.4.1. Fluorescence Resonance Energy Transfer (FRET) and Excimer Formation

Linear binary probes are widely used for RNA imaging [136] including for FRET approach. For monitoring the c-*fos* gene mRNA in living COS7 cells before and after porbol-12-myristate-13-acetate (PMA) stimulation, binary probes based on cyanine-labeled oligo(2'-O-methylribonucleotides) and FRET approach were used [217]. Cy3 and Cy5 dyes were taken as donor and acceptor, respectively. The authors managed to demonstrate the increase of FRET signal after PMA stimulation. 

An approach similar to FRET is based on the property of two pyrene residues to form excimers being in a close proximity [131]. When two oligonucleotides directed against adjacent target RNA sequences are labeled with pyrene (at 3'- and 5'-end, respectively), strong excimer emission appears upon their hybridization with the target. One of the most interesting examples is the work of Martí *et al*. [218] in cell extracts. They used two oligo(2'-O-methylribonucleotides) labeled by pyrene at 3'- and 5'-end, respectively. Upon hybridization with their target these probes formed a tandem in which two pyrenes were located in a close proximity and formed an excimer. The advantages of the pyrene probes are the following: high Stokes shift between monomer and excimer emission, and their relatively long lifetime (30–60 ns) compared to the lifetime of the autofluorescence background (about 8 ns). The authors used time-resolved emission spectroscopy in order to observe only excimer emission. This method has been applied for detection of sensorin mRNA in cell extracts from *A. californica* pleural ganglia.

#### 4.4.2. Template-Directed Chemical Reactions with Activation or Formation of a Fluorophore

**Figure 16:**
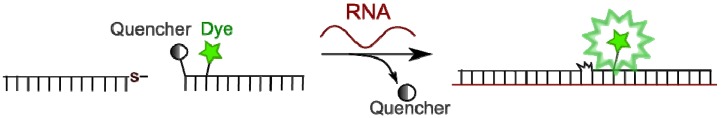
Quenched autoligation probes [173].

Another approach is to use a specific template-directed chemical reaction to generate a fluorophore-binding structure (or to initiate fluorophore synthesis in a template-directed reaction) using two functional groups that are attached to each component of a binary probe and become adjacent upon interaction with a target. Kool’s group has developed quenched autoligation (QUAL) probes [219] (Figure 16).

One component of the probe contains a 3'-thiophosphate group; the other one contains fluorescein and quencher dabsyl at the same terminus. No fluorescence is observed without target. Dabsyl is attached via activated hydroxyl group that is able to react with the thiophosphate group. When two components are in a close proximity on the target, a reaction between thiophosphate and dabsyl-activated hydroxyl leads to the autoligation of two probes and the following release of dabsyl into the medium [220]. If the new linker between two components is a relatively long hydrocarbon chain, it destabilizes the ligated complementary complex, the ligation product dissociates from the target that becomes again available for the next cycle. Accumulation of fluorescent ligation products leads to amplification of the signal [221]. This method has also been used in a FRET version [222,223]. The cyanine acceptor Cy5 was inserted into the thiophosphate component of the binary probe. Energy transfer from fluorescein to Cy5 after matrix-assisted ligation leads to a higher intensity of the fluorescent signal and enhances the sensitivity of the probe. The use of oligo(2'-O-methylribonucleotides) as recognition units provides higher hybridization rates and better stability [222]. The QUAL probes have been applied for live cell imaging [220,221,222,223,224], for example, for discrimination between three closely related bacteria using slight differences in their ribosomal RNAs [223,224].

Further development of the binary template-catalyzed probes is related to new chemistries [225,226]. Covalent ligation of two components increases affinity of a probe to its target. When reaction proceeds without ligation, dissociation of the probes is facilitated; that opens the way for catalytic reactions with multiple turnovers and hence signal amplification. Kool *et al*. proposed to use a reductive quencher release by bioortogonal template-directed Staudinger reaction [226]. The first component of the binary probe contains a fluorescein residue and a quencher attached via α-azidoester at the same terminus. The other component contains triphenylphosphine. Reductive release of the quencher due to the Staudinger reaction induces green fluorescence. After dissociation of both components from the template, new components associate and reaction proceeds again in multiple turnovers. A FRET version of this binary system with fluorescein and TAMRA has also been realized [226]. Further developments of this reaction, such as sandwich probes (three linear components and two simultaneous quencher release reactions) [227], system with one fluorophore and two quenchers at the 3'-terminus of the first component and their release via two consecutive reductions by two triphenylphosphine moieties at the 5'-terminus of the second component [228] or two-color binary probes for fine discrimination of rRNAs possessing nearly identical sequences in bacteria [229] can be cited. They largely improve selectivity and sensitivity of the detection, decrease background signal and provide high fluorescence turn-on ratios.

**Figure 17:**
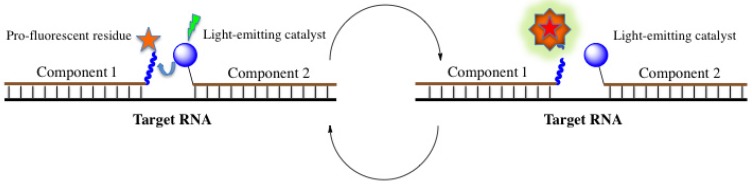
Template-directed light-induced catalyzed cleavage of a linker in pro-fluorophore-conjugated probe with the release of fluorescent molecule.

A similar approach has been proposed by Winssinger’s team. They use slightly different technique of catalytic release of quenched pro-fluorophore, which becomes fluorescent due to rearrangement of its structure. The idea was to attach this pro-fluorophore via photolabile linkage to the first oligonucleotide component and to cleave this linkage on the target template by energy transfer from the second light-activated fluorophore attached to the second oligonucleotide component [230] (Figure 17). 

In their recent publications, the authors used PNA oligomers as template-binding parts of the probes and ruthenium-catalyzed azide photoreduction in the presence of sodium ascorbate as a fluorophore-releasing reaction [231]. To realize this approach, they attached to the first PNA molecule pro-fluorescent rhodamine residue linked through an immolative linker containing azido-group. The second PNA was conjugated to ruthenium (II) complex: bis(2,20-bipyridine)-(5-isothiocyanato-phenanthroline) ruthenium. Ru(II)-catalyzed reduction of azido group on the target template under visible light irradiation leads to cleavage of the linker and release of the fluorescent rhodamine form. Multiple turnover of the catalytic reaction provides signal amplification. The authors successfully used these probes for specific imaging of microRNAs in permeabilized or transfected HeLa and breast cancer living cells [232]. 

Seitz *et al*. used a similar principle but different chemistry: iso-cystein-mediated transfer reaction [233]. They managed to transfer a quencher dabsyl from fluorescein-labeled PNA probe to TAMRA-labeled one on the target template, thus changing the signal color from red to green. Combining this method with ELISA for further signal amplification, they could detect 500 attomoles of HIV-I RNA *in vitro* [234]*.* Further improvement of the method was achieved by a direct synthesis of the fluorescence molecule *de novo* on the target template [235]. Two PNA oligonucleotides bearing two “halves” of stilbene fluorophore “meet” on the template. One of these “halves” is PNA-tethered benzaldehyde, the other one is 6-cyanobensyl moiety attached to PNA via triphenylphosphonium salt. Wittig reaction between two components leads to formation of stilbene residue attached to the first PNA with the cleavage of the linkage between second “half” and second PNA. Stilbene fluorescence additionally enhances by complex formation with its receptor α-cyclodextrin. Finally, the fluorescence enhancement reached 300 times. However, on our knowledge, these systems were never used in fixed or living cells. 

#### 4.4.3. Aptamers as Binary Probes

**Figure 18:**
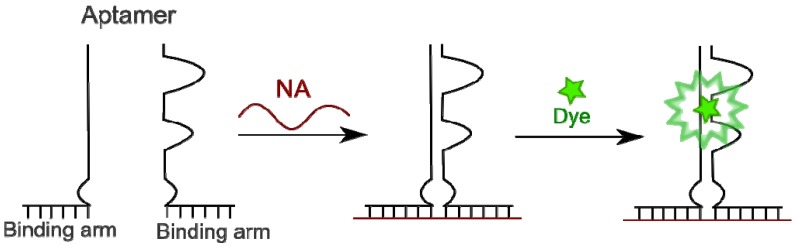
Fluorophore-binding aptamers as binary probes.

Aptamers are artificially selected nucleic acids possessing high binding affinity to certain molecules or structures, such as different small molecules (e.g., dyes, drugs, biologically active substances, organic molecules, *etc*.), proteins, peptides, nucleic acids and many other targets (for recent reviews, see [236,237,238]). The aptamer approach is a part of a more general combinatorial strategy [239]. The advantage of RNA or DNA aptamers is, on one hand, a possibility to attach to their ends any nucleotide sequence complementary to a target RNA for matrix binding. On the other hand, it is possible to split the whole aptamer strand in two parts. Each of them is separately able to recognize a target but unable to recognize a substrate. Template-directed assembly of the aptamers on the target RNA due to interactions with the adjacent complementary sequences leads to the folding into its native structure that recovers substrate-binding ability. If this molecule is a fluorophore, especially one that can modulate its fluorescence properties upon aptamer binding, an enhanced signal is observed on the target nucleic acid only when both parts of the aptamer are bound to the adjacent positions. The principle of the method was demonstrated by targeting single-stranded DNA molecules with a malachite green-binding RNA aptamer [240], and with a DNA aptamer recognizing a Hoechst derivative modified by two *tert*-butyl groups to abrogate its binding to A-T regions in the DNA minor groove [241]. In both cases, a strong light-up effect has been observed upon interaction of the substrate dye with template-assembled aptamer (Figure 18). 

This principle opens a way for single-stranded RNA imaging *in cellulo*: as it is mentioned in [240], aptamers can be expressed directly in the living cells from engineered vectors. Indeed, applications of this principle to RNA detection have been shown. An aptamer selected against sulforodamine was used for detection of target RNAs in cell extracts but not in living cells [242]. Design of aptamers that are able to bind synthetic molecules mimicking the fluorophore center of GFP or enhanced GFP (EGFP) and to activate fluorescence have been described by Paige *et al*. [243]. These fluorophore-aptamer complexes were used as RNA analogs of GFP and EGFP in living cells. For more information on the aptamer probing, we address the reader to recent reviews on the subject [135,136,244].

### 4.5. Modified Oligonucleotides in Design of Nucleic Acid Probes

**Figure 19:**
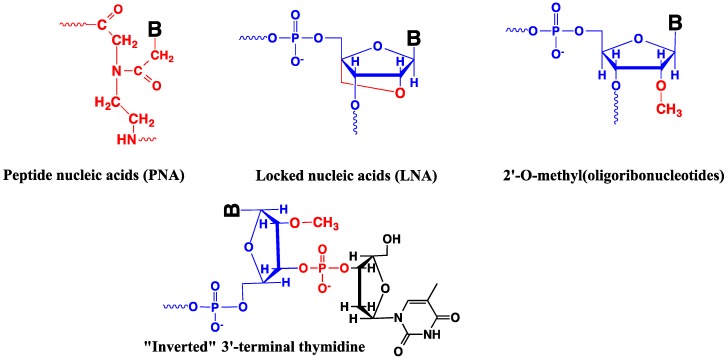
Modified oligonucleotides used for probe design. B—nucleobase. Modifications are indicated in red.

Oligonucleotides (including linear probes, molecular beacons, binary probes and aptamers) are highly specific to target sequences. The use of binary probes and molecular beacons opens a way to increase their sensitivity and signal-to-background ratio. However, the common drawbacks of oligonucleotide probes are (i) their poor stability in biological media (that is especially true for RNA), and (ii) their low penetration into living cells. Concerning instability, modified oligonucleotides can be used to circumvent this problem. The most adapted for this purpose seem to be PNA [46,47,230,231,232,233,234,235], LNA [27,28,29,211,212,213,214,245] and 2'-O-methyl-RNA [132,133,134,163,186,189,215,217,218,220,222,223,224] (Figure 19). According to literature, one of the most suitable targeting oligonucleotide analogs that respond to a majority of requirements are oligo(2'-O-methylribonucleotides). They are more resistant in biological media than natural RNA and form relatively stable complementary complexes with DNA and particularly with RNA, as well as triple helices with double-stranded DNA [246,247,248]. Coupling of thymidine nucleotide to 3'-terminus of the probe via “inverted” 3'-3'-phosphodiester bonds (Figure 19) stabilizes modified oligonucleotides against 3'-exonucleases [249,250,251]. Oligo(2'-O-methylribonucleotides) exhibit high rates of hybridization with nucleic acid targets, increased ability to bind structured RNA regions and to discriminate nucleotide mismatches in duplexes with RNA and DNA [252]. In addition, duplexes of oligo(2'-O-methylribonucleotides) with DNA are not substrates for RNase H. They are not cleaved by this enzyme [253], in contrast to RNA. They are not substrates of DNA or RNA polymerases and can be used directly in biological systems in the presence of these enzymes. 

## 5. Intracellular Delivery of Oligonucleotides and Their Analogs

In majority of cases, the transport of probes for DNA or RNA detection into the living cells is a serious problem. It especially affects nucleic acid probes or engineered vectors. For intracellular delivery of oligonucleotides or plasmids bearing genes of endogenous RNA, aptamers or fused fluorescent proteins, a wide range of methods can be used: transfection with cationic lipids or liposomes, cationic polymers and dendrimers, peptide vectors (cell-penetrating peptides), nanoparticles, as well as microinjection, electroporation, mild detergent or pore-forming toxin streptolysin-O treatment, viral vectors, *etc*. [254,255,256,257]. Development of non-toxic, low-invasive transporters for intracellular probe delivery is a large field of molecular biology, bionanotechnology and nanomedicine that could be a subject of separate review. Among the recently designed delivery vectors that have a promising future, the carbon nanotubes are worth mentioning [258,259,260,261,262,263]. 

## 6. Conclusions

Fluorescent microscopy of intracellular nucleic acids made great progress during the last decades. Despite the fact that the greater part of information about cellular localization of DNA and RNA has been obtained by the FISH method in fixed cells, increasing attention is now paid to direct dynamic imaging in living cells. It is a promising field of research still in full development. 

In case of native DNA, different probes can be used, such as colored fluorescent proteins fused with DNA-binding proteins and expressed directly in the cells (the most sequence-specific being zinc fingers and TALEs), triplex-forming oligonucleotides and polyamide sequence-specific minor groove binders. In addition, labeled modified oligonucleotides (as LNA, and PNA) can be employed, but their interaction with dsDNA assumes its local denaturation and strand displacement. Among all these probes, only polyamide minor groove binders can penetrate into living cells without transporters. However, several problems, such as relatively low sequence specificity, insufficient sensitivity for unique sequences and high fluorescent background in living cells, still limit their potential applications. These problems could be overtaken by modifying MGB structures, selecting new light-up fluorophores and developing binary probes adapted for dsDNA.

In the case of RNA, despite development of engineered systems with fused fluorescent and RNA-binding proteins, only nucleic acid probes and their modified analogs can provide sequence-specific and sensitive RNA detection and visualization. Except for bad cell penetration, the problem of high background in living cells also arises. Several approaches are proposed to resolve this issue. Molecular beacons and different binary probes, such as FRET pairs, template-directed fluorophore activation or synthesis, excimers and exciplexes, fluorophore-binding aptamers and other systems have largely contributed toward solutions to this matter.

Penetration of labeled probes into living cells without cytotoxicity and side effects affecting cell functioning is a separate and very important task that also has to be considered during the design of reagents for live cell probing.

## Abbreviations

BHQ: black hole quencher
BO, Cy3, Cy5, DRAQ5, TAMRA, TOTO: commercial names of fluorophores
DAPI: 4',6-diamidino-2-phenylindole
ELISA: enzyme-linked immunosorbent assay
FRET: fluorescence resonance energy transfer
GFP: green fluorescent protein
FISH: fluorescence *in situ* hybridization
LNA: locked nucleic acid
MGB: minor groove binder
PCR: polymerase chain reaction
PET: positron emission tomography
PNA: peptide nucleic acid
QUAL: quenched autoligation probe
SSB: single strand DNA-binding protein
TALE: transcription activator-like effector
TALEN: artificial nuclease based on TALE
TFO: triplex-forming oligonucleotide
TINA: twisted intercalating nucleic acid
TISH: triplex *in situ* hybridization
TO: thiazole orange
TR: thiazole red

## References

[1] Dirks R.W., Tanke H.J. (2006). Advances in fluorescent tracking of nucleic acids in living cells. BioTechniques.

[2] Silverman A.P., Kool E.T. (2005). Quenched probes for highly specific detection of cellular RNAs. Trends Biotechnol..

[3] Weigert R., Porat-Shliom N., Amornphimoltham P. (2013). Imaging cell biology in live animals: Ready for prime time. J. Cell Biol..

[4] Soon W.W., Hariharan M., Snyder M.P. (2013). High-throughput sequencing for biology and medicine. Mol. Syst. Biol..

[5] Bernstein B.E., Birney E., Dunham I., Green E.D., Gunter C., Snyder M. (2012). An integrated encyclopedia of DNA elements in the human genome. Nature.

[6] Klein K., Gigler A.M., Aschenbrenne T., Monetti R., Bunk W., Jamitzky F., Morfill G., Stark R.W., Schlegel J. (2012). Label-free live-cell imaging with confocal Raman microscopy. Biophys. J..

[7] Palonpon A.F., Ando J., Yamakoshi H., Dodo K., Sodeoka M., Kawata S., Fujita K. (2013). Raman and SERS microscopy for molecular imaging of live cells. Nat. Protoc..

[8] Lendvai G., Estrada S., Bergstrom M. (2009). Radiolabelled oligonucleotides for imaging of gene expression with PET. Curr. Med. Chem..

[9] Harki D.A., Satyamurthy N., Stout D.B., Phelps M.E., Dervan P.B. (2008). *In vivo* imaging of pyrrole-imidazole polyamides with positron emission tomography. Proc. Nat. Acad. Sci. USA.

[10] Fernández-Suárez M., Ting A.Y. (2008). Fluorescent probes for super-resolution imaging in living cells. Nat. Rev. Mol. Cell. Biol..

[11] Tsien R.Y. (1998). The geen fluorescent protein. Annu. Rev. Biochem..

[12] Heim R., Tsien R.Y. (1996). Engineering green fluorescent protein for improved brightness, longer wavelengths and fluorescence resonance energy transfer. Curr. Biol..

[13] Zimmer M. (2002). Green fluorescent protein (GFP):  Applications, structure, and related photophysical behavior. Chem. Rev..

[14] Lippincott-Schwartz J., Patterson G.H. (2003). Development and use of fluorescent protein markers in living cells. Science.

[15] Choo K.H.A. (1997). The Centromere.

[16] Oulton R., Harrington L. (2000). Telomeres, telomerase, and cancer: Life on the edge of genomic stability. Curr. Opin. Oncol..

[17] Rezler E.M., Bearss D.J., Hurley L.H. (2003). Telomere inhibition and telomere disruption as processes for drug targeting. Annu. Rev. Pharmacol. Toxicol..

[18] O'Sullivan R.J., Karlseder J. (2010). Telomeres: Protecting chromosomes against genome instability. Nat. Rev. Mol. Cell. Biol..

[19] Nath J., Johnson K.L. (2000). A review of Fluorescence *in Situ* Hybridization (FISH): Current status and future prospects. Biotech. Histochem..

[20] Levsky J.M., Singer R.H. (2003). Fluorescence *in Situ* hybridization: Past, present and future. J. Cell Sci..

[21] Smolina I., Lee C., Frank-Kamenetskii M. (2007). Detection of low-copy-number genomic DNA sequences in individual bacterial cells by using peptide nucleic acid-assisted rolling-circle amplification and fluorescence *in situ* hybridization. Appl. Environ. Microbiol..

[22] Jaco I., Canela A., Vera E., Blasco M.A. (2008). Centromere mitotic recombination in mammalian cells. J. Cell Biol..

[23] Paulasova P., Pellestor F. (2004). The peptide nucleic acids (PNAs): A new generation of probes for genetic and cytogenetic analyses. Ann. Genet. Paris.

[24] Pellestor F., Paulasova P. (2004). The peptide nucleic acids, efficient tools for molecular diagnosis (Review). Int. J. Mol. Med..

[25] Pellestor F., Paulasova P. (2004). The peptide nucleic acids (PNAs), powerful tools for molecular genetics and cytogenetics. Eur. J. Hum. Genet..

[26] Astakhova I.V., Ustinov A.V., Korshun V.A., Wengel J. (2011). LNA for optimization of fluorescent oligonucleotide probes: Improved spectral properties and target binding. Bioconjug. Chem..

[27] Silahtaroglu A., Pfundheller H., Koshkin A., Tommerup N., Kauppinen S. (2004). LNA-modified oligonucleotides are highly efficient as FISH probes. Cytogenet. Genome Res..

[28] Silahtaroglu A.N., Tommerup N., Vissing H. (2003). FISHing with locked nucleic acids (LNA): Evaluation of different LNA/DNA mixmers. Mol. Cell Probes.

[29] Thomsen R., Nielsen P.S., Jensen T.H. (2005). Dramatically improved RNA *in situ* hybridization signals using LNA-modified probes. RNA.

[30] Bridger J.M., Volpi E.V., Schmitt E., Schwarz-Finsterle J., Stein S., Boxler C., Müller P., Mokhir A., Krämer R., Cremer C. (2010). Combinatorial Oligo FISH: Directed Labeling of Specific Genome Domains in Differentially Fixed Cell Material and Live Cells. Methods in Molecular Biology: Fluorescence in situ Hybridization (FISH).

[31] Hausmann M., Winkler R., Hildenbrand G., Finsterle J., Weisel A., Rapp A., Schmitt E., Janz S., Cremer C. (2003). COMBO-FISH: Specific labeling of nondenatured chromatin targets by computer-selected DNA oligonucleotide probe combinations. BioTechniques.

[32] Schwarz-Finsterle J., Stein S., Grossmann C., Schmitt E., Trakhtenbrot L., Rechavi G., Amariglio N., Cremer C., Hausmann M. (2007). Comparison of triple helical COMBO-FISH and standard FISH by means of quantitative microscopic image analysis of ABL/BCR positions in cell nuclei. J. Biochem. Biophys. Methods.

[33] Stevens N., O’Connor N., Vishwasrao H., Samaroo D., Kandel E.R., Akins D.L., Drain C.M., Turro N.J. (2008). Two Color RNA intercalating probe for cell imaging applications. J. Am. Chem. Soc..

[34] Martin R.M., Leonhardt H., Cardoso M.C. (2005). DNA labeling in living cells. Cytometry Part A.

[35] Kapuscinski J. (1995). DAPI: A DNA-specific fluorescent probe. Biotech. Histochem..

[36] Pljevaljčić G., Pignot M., Weinhold E. (2003). Design of a new fluorescent cofactor for DNA methyltransferases and sequence-specific labeling of DNA. J. Am. Chem. Soc..

[37] Pljevaljcic G., Schmidt F., Peschlow A., Weinhold E. (2004). Sequence-specific DNA labeling using methyltransferases. Methods Mol. Biol..

[38] Pljevaljčić G., Schmidt F., Scheidig A.J., Lurz R., Weinhold E. (2007). Quantitative labeling of long plasmid DNA with nanometer precision. ChemBioChem.

[39] Maya-Mendoza A., Olivares-Chauvet P., Kohlmeier F., Jackson D.A. (2012). Visualising chromosomal replication sites and replicons in mammalian cells. Methods.

[40] Brickner D.G., Light W., Brickner J.H., Jonathan W., Christine G., Gerald R.F. (2010). Chapter 22—Quantitative Localization of Chromosomal Loci by Immunofluorescence. Methods in Enzymology.

[41] Biffi G., Tannahill D., McCafferty J., Balasubramanian S. (2013). Quantitative visualization of DNA G-quadruplex structures in human cells. Nat. Chem..

[42] Sugimoto K., Senda-Murata K., Oka S. (2008). Construction of three quadruple-fluorescent MDA435 cell lines that enable monitoring of the whole chromosome segregation process in the living state. Mutat. Res. Genet. Toxicol. Environ. Mutagen..

[43] Wang X., Reyes-Lamothe R., Sherratt D.J. (2008). Visualizing genetic loci and molecular machines in living bacteria (Biochemical Society Linked Focused Meetings). Biochem. Soc. Trans..

[44] De Vos W.H., Hoebe R.A., Joss G.H., Haffmans W., Baatout S., van Oostveldt P., Manders E.M.M. (2009). Controlled light exposure microscopy reveals dynamic telomere microterritories throughout the cell cycle. Cytom. Part A.

[45] De Vos W.H., Joss G.H., Haffmans W., Hoebe R.A., Manders E.M.M., van Oostveldt P. (2010). Four-dimensional telomere analysis in recordings of living human cells acquired with Controlled Light Exposure Microscopy. J. Microsc..

[46] Molenaar C., Wiesmeijer K., Verwoerd N.P., Khazen S., Eils R., Tanke H.J., Dirks R.W. (2003). Visualizing telomere dynamics in living mammalian cells using PNA probes. EMBO J..

[47] Flierl A., Jackson C., Cottrell B., Murdock D., Seibel P., Wallace D.C. (2003). Targeted delivery of DNA to the mitochondrial compartment via import sequence-conjugated peptide nucleic acid. Mol. Ther..

[48] Garvie C.W., Wolberger C. (2001). Recognition of specific DNA sequences. Mol. Cell.

[49] Tachikawa K., Briggs S.P. (2006). Targeting the human genome. Curr. Opin. Biotechnol..

[50] Ghosh I., Stains C.I., Ooi A.T., Segal D.J. (2006). Direct detection of double-stranded DNA: Molecular methods and applications for DNA diagnostics. Mol. Biosyst..

[51] Rodriguez-Martinez J.A., Peterson-Kaufman K.J., Ansari A.Z. (2010). Small-molecule regulators that mimic transcription factors. Biochim. Biophys. Acta-Gene Regul. Mech..

[52] Klug A. (2010). The discovery of zinc fingers and their applications in gene regulation and genome manipulation. Annu. Rev. Biochem..

[53] Urnov F.D., Rebar E.J., Holmes M.C., Zhang H.S., Gregory P.D. (2010). Genome editing with engineered zinc finger nucleases. Nat. Rev. Genet..

[54] Lindhout B.I., Fransz P., Tessadori F., Meckel T., Hooykaas P.J.J., van der Zaal B.J. (2007). Live cells imaging of repetitive DNA sequences via GFP-tagged polydactyl zinc finger proteins. Nucleic Acids Res..

[55] Boch J., Scholze H., Schornack S., Landgraf A., Hahn S., Kay S., Lahaye T., Nickstadt A., Bonas U. (2009). Breaking the code of DNA binding specificity of TAL-type III effectors. Science.

[56] Mak A.N.-S., Bradley P., Cernadas R.A., Bogdanove A.J., Stoddard B.L. (2012). The crystal structure of TAL effector PthXo1 bound to its DNA target. Science.

[57] Christian M., Cermak T., Doyle E.L., Schmidt C., Zhang F., Hummel A., Bogdanove A.J., Voytas D.F. (2010). Targeting DNA double-strand breaks with TAL effector nucleases. Genetics.

[58] Miller J.C., Tan S., Qiao G., Barlow K.A., Wang J., Xia D.F., Meng X., Paschon D.E., Leung E., Hinkley S.J. (2011). A TALE nuclease architecture for efficient genome editing. Nat. Biotech..

[59] Bedell V.M., Wang Y., Campbell J.M., Poshusta T.L., Starker C.G., Krug Ii R.G., Tan W., Penheiter S.G., Ma A.C., Leung A.Y.H. (2012). *In vivo* genome editing using a high-efficiency TALEN system. Nature.

[60] Praseuth D., Guieysse A.L., Hélène C. (1999). Triple helix formation and the antigene strategy for sequence-specific control of gene expression. Biochim. Biophys. Acta Gene Struct. Express..

[61] Faria M., Giovannangeli C. (2001). Triplex-forming molecules: From concepts to applications. J. Gene Med..

[62] Dervan P.B., Bürli R.W. (1999). Sequence-specific DNA recognition by polyamides. Curr. Opin. Chem. Biol..

[63] Dervan P.B. (2001). Molecular recognition of DNA by small molecules. Bioorgan. Med. Chem..

[64] Sun J.S., Hélène C. (1993). Oligonucleotide-directed triple-helix formation. Curr. Opin. Struct. Biol..

[65] Fox K.R. (2000). Targeting DNA with triplexes. Curr. Med. Chem..

[66] Casey B.P., Glazer P.M., Moldave K. (2001). Gene Targeting via Triple-Helix Formation. Progress in Nucleic Acid Research and Molecular Biology.

[67] Knauert M.P., Glazer P.M. (2001). Triplex forming oligonucleotides: Sequence-specific tools for gene targeting. Hum. Mol. Genet..

[68] Vasquez K.M., Glazer P.M. (2002). Triplex-forming oligonucleotides: Principles and applications. Quart. Rev. Biophys..

[69] Duca M., Vekhoff P., Oussedik K., Halby L., Arimondo P.B. (2008). The triple helix: 50 years later, the outcome. Nucleic Acids Res..

[70] Mergny J.L., Boutorine A.S., Garestier T., Belloc F., Rougée M., Bulychev N.V., Koshkin A.A., Bourson J., Lebedev A.V., Valeur B. (1994). Fliorescence energy transfer as a probe for nucleic acid structure and sequence. Nucleic Acids Res..

[71] Grimm G.N., Boutorine A.S., Lincoln P., Nordén B., Hélène C. (2002). Formation of DNA triple helices by an oligonucleotide conjugated to a fluorescent ruthenium complex. ChemBioChem.

[72] Filichev V.V., Pedersen E.B. (2005). Stable and selective formation of Hoogsteen-type triplexes and duplexes using twisted intercalating nucleic acids (TINA) prepared via postsynthetic sonogashira solid-phase coupling reactions. J. Am. Chem. Soc..

[73] Filichev V.V., Nielsen M.C., Bomholt N., Jessen C.H., Pedersen E.B. (2006). High thermal stability of 5'-5'-linked alternate Hoogsteen triplexes at physiological pH. Angew. Chem. Int. Ed..

[74] Géci I., Filichev V., Pedersen E. (2007). Stabilization of parallel triplexes by Twisted Intercalating Nucleic Acids (TINAs) Incorporating 1,2,3-triazole units and prepared by microwave-accelerated click chemistry. Chem. Eur. J..

[75] Doluca O., Boutorine A.S., Filichev V.V. (2011). Triplex-forming Twisted Intercalating Nucleic Acids (TINAs): Design rules, stabilization of antiparallel DNA triplexes and inhibition of G-quartet-dependent self-association. ChemBioChem.

[76] Van Daele I., Bomholt N., Filichev V.V., van Calenbergh S., Pedersen E.B. (2008). Triplex formation by pyrene-labelled probes for nucleic acid detection in fluorescence assays. ChemBioChem.

[77] Renard B.L., Lartia R., Asseline U. (2008). Targeting DNA with “light-up” pyrimidine triple-helical forming oligonucleotides conjugated to stabilizing fluorophores (LU-TFOs). Org. Biomol. Chem..

[78] Johnson M.D., Fresco J.R. (1999). Third-strand *in situ* hybridization (TISH) to non-denatured metaphase spreads and interphase nuclei. Chromosoma.

[79] Dervan P.B., Edelson B.S. (2003). Recognition of the DNA minor groove by pyrrole-imidazole polyamides. Curr. Opin. Struct. Biol..

[80] Heckel A., Dervan P.B. (2003). U-Pin polyamide motif for recognition of the DNA minor groove. Chem. Eur. J..

[81] Farkas M.E., Tsai S.M., Dervan P.B. (2007). α-Diaminobutyric acid-linked hairpin polyamides. Bioorg. Med. Chem..

[82] Meier J.L., Montgomery D.C., Dervan P.B. (2012). Enhancing the cellular uptake of Py-Im polyamides through next-generation aryl turns. Nucleic Acids Res..

[83] Marques M.A., Doss R.M., Foister S., Dervan P.B. (2004). Expanding the repertoire of heterocycle ring pairs for programmable minor groove DNA recognition. J. Am. Chem. Soc..

[84] Krutzik P.O., Chamberlin A.R. (2002). Rapid solid-phase synthesis of DNA-binding pyrrole-imidazole polyamides. Bioorg. Med. Chem. Lett..

[85] Krutzik P.O., Chamberlin A.R. (2002). Synthesis of DNA-binding polyamides. Robust solid-phase methods for coupling heterocyclic aromatic amino acids. Methods Mol. Biol..

[86] Chenoweth D.M., Harki D.A., Dervan P.B. (2009). Solution-phase synthesis of pyrrole-imidazole polyamides. J. Am. Chem. Soc..

[87] Burnett R., Melander C., Puckett J.W., Son L.S., Wells R.B., Dervan P.B., Gottesfeld J.M. (2006). DNA sequence-specific polyamides alleviate transcription inhibition associated with long GAA center dot TTC repeats in Friedreich’s ataxia. Proc. Nat. Acad. Sci. USA.

[88] Dudouet B., Burnett R., Dickinson L.A., Wood M.R., Melander C., Belitsky J.M., Edelson B., Wurtz N., Briehn C., Dervan P.B. (2003). Accessibility of nuclear chromatin by DNA binding polyamides. Chem. Biol..

[89] Nickols N.G., Dervan P.B. (2007). Suppression of androgen receptor-mediated gene expression by a sequence-specific DNA-binding polyamide. Proc. Nat. Acad. Sci. USA.

[90] Nickols N.G., Jacobs C.S., Farkas M.E., Dervan P.B. (2007). Improved nuclear localization of DNA-binding polyamides. Nucleic Acids Res..

[91] Dervan P.B., Doss R.M., Marques M.A. (2005). Programmable DNA binding oligomers for control of transcription. Curr. Med. Chem. Anti-Cancer Agents.

[92] Szewczyk J.W., Baird E.E., Dervan P.B. (1996). Sequence-specific recognition of DNA by a major and minor groove binding ligands. Angew. Chem. Int. Ed..

[93] Szewczyk J.W., Baird E.E., Dervan P.B. (1996). Cooperative triple-helix formation via a minor groove dimerization domain. J. Am. Chem. Soc..

[94] Trauger J.W., Baird E.E., Dervan P.B. (1998). Recognition of 16 base pairs in the minor groove of DNA by a pyrrole-imidazole polyamide dimer. J. Am. Chem. Soc..

[95] Herman D.M., Baird E.E., Dervan P.B. (1999). Tandem hairpin motif for recognition in the minor groove of DNA by pyrrole - imidazole polyamides. Chem. Eur. J..

[96] Kers I., Dervan P.B. (2002). Search for the optimal linker in tandem hairpin polyamides. Bioorg. Med. Chem..

[97] Halby L., Ryabinin V.A., Sinyakov A.N., Boutorine A.S. (2005). Functionalized head-to-head hairpin polyamides: Synthesis, double-stranded DNA-binding activity and affinity. Bioorg. Med. Chem. Lett..

[98] Bhattacharya S., Thomas M. (2002). DNA binding properties of novel dansylated distamycin analogues in which the fluorophore is directly conjugated to the N-methylpyrrole carboxamide backbone. J. Biomol. Struct. Dyn..

[99] Rucker V.C., Foister S., Melander C., Dervan P.B. (2003). Sequence-specific fluorescence detection of double strand DNA. J. Am. Chem. Soc..

[100] Fechter E.J., Olenyuk B., Dervan P.B. (2005). Sequence-specific fluorescence detection of DNA by polyamide-thiazole orange conjugates. J. Am. Chem. Soc..

[101] Bando T., Fujimoto J., Minoshima M., Shinohara K.I., Sasaki S., Kashiwazaki G., Mizumura M., Sugiyama H. (2007). Detection of CAG repeat DNA sequences by pyrene-functionalized pyrrole-imidazole polyamides. Bioorg. Med. Chem..

[102] Chenoweth D.M., Viger A., Dervan P.B. (2007). Fluorescent sequence-specific dsDNA binding oligomers. J. Am. Chem. Soc..

[103] Fujimoto J., Bando T., Minoshima M., Kashiwazaki G., Nishijima S., Shinohara K., Sugiyama H. (2008). Perylene-conjugated pyrrole polyamide as a sequence-specific fluorescent probe. Bioorg. Med. Chem..

[104] Hsu C.F., Dervan P.B. (2008). Quantitating the concentration of Py-Im polyamide-fluorescein conjugates in live cells. Bioorg. Med. Chem. Lett..

[105] Nishijima S., Shinohara K., Bando T., Minoshima M., Kashiwazaki G., Sugiyama H. (2010). Cell permeability of Py-Im-polyamide-fluorescein conjugates: Influence of molecular size and Py/Im content. Bioorg. Med. Chem..

[106] Dupureur C.M., Bashkin J.K., Aston K., Koeller K.J., Gaston K.R., He G. (2012). Fluorescence assay of polyamide-DNA interactions. Anal. Biochem..

[107] Maeshima K., Janssen S., Laemmli U.K. (2001). Specific targeting of insect and vertebrate telomeres with pyrrole and imidazole polyamides. EMBO J..

[108] Vaijayanthi T., Bando T., Pandian G.N., Sugiyama H. (2012). Progress and prospects of pyrrole-imidazole polyamide–fluorophore conjugates as sequence-selective DNA probes. ChemBioChem.

[109] Dickinson L.A., Burnett R., Melander C., Edelson B.S., Arora P.S., Dervan P.B., Gottesfeld J.M. (2004). Arresting cancer proliferation by small-molecule gene regulation. Chem. Biol..

[110] Peng X., Wu T., Fan J., Wang J., Zhang S., Song F., Sun S. (2011). An effective minor groove binder as a red fluorescent marker for live-cell DNA imaging and quantification. Angew. Chem. Int. Ed..

[111] Melander C., Burnett R., Gottesfeld J.M. (2004). Regulation of gene expression with pyrrole-imidazole polyamides. J. Biotechnol..

[112] Minoshima M., Chou J.C., Lefebvre S., Bando T., Shinohara K., Gottesfeld J.M., Sugiyama H. (2010). Potent activity against K562 cells by polyamide-seco-CBI conjugates targeting histone H4 genes. Bioorg. Med. Chem..

[113] Jacobs C.S., Dervan P.B. (2009). Modifications at the C-terminus to improve pyrrole-imidazole polyamide activity in cell culture. J. Med. Chem..

[114] Synold T.W., Xi B.X., Wu J., Yen Y., Li B.C., Yang F., Phillips J.W., Nickols N.G., Dervan P.B. (2012). Single-dose pharmacokinetic and toxicity analysis of pyrrole-imidazole polyamides in mice. Cancer Chemother. Pharmacol..

[115] Raskatov J.A., Hargrove A.E., So A.Y., Dervan P.B. (2012). Pharmacokinetics of Py-Im polyamides depend on architecture: Cyclic *versus* linear. J. Am. Chem. Soc..

[116] Kawamoto Y., Bando T., Kamada F., Li Y., Hashiya K., Maeshima K., Sugiyama H. (2013). Development of a new method for synthesis of tandem hairpin pyrrole–imidazole polyamide probes targeting human telomeres. J. Am. Chem. Soc..

[117] Svanvik N., Nygren J., Westman G., Kubista M. (2001). Free-probe fluorescence of light-up probes. J. Am. Chem. Soc..

[118] Svanvik N., Westman G., Wang D., Kubista M. (2000). Light-Up Probes: Thiazole orange-conjugated peptide nucleic acid for detection of target nucleic acid in homogeneous solution. Anal. Biochem..

[119] Kricka L.J. (2002). Stains, labels and detection strategies for nucleic acids assays. Ann. Clin. Biochem..

[120] Hilal H., Taylor J.A. (2008). Cyanine dyes for the detection of double stranded DNA. J. Biochem. Biophys. Meth..

[121] Cullander C. (1994). Imaging in the far-red with electronic light microscopy: Requirements and limitations. J. Microsc..

[122] Ohulchanskyy T.Y., Pudavar H.E., Yarmoluk S.M., Yashchuk V.M., Bergey E.J., Prasad P.N. (2003). A monomethine cyanine dye Cyan 40 for two-photon-excited fluorescence detection of nucleic acids and their visualization in live cells. Photochem. Photobiol..

[123] Akbay N., Losytskyy M., Kovalska V., Balanda A., Yarmoluk S. (2008). The mechanism of benzothiazole styrylcyanine dyes binding with dsDNA: Studies by spectral-luminescent methods. J. Fluoresc..

[124] Losytskyy M.Y., Volkova K.D., Kovalska V.B., Makovenko I.E., Slominskii Y.L., Tolmachev O.I., Yarmoluk S.M. (2005). Fluorescent properties of pentamethine cyanine dyes with cyclopentene and cyclohexene group in presence of biological molecules. J. Fluoresc..

[125] Kovalska V.B., Kryvorotenko D.V., Balanda A.O., Losytskyy M.Y., Tokar V.P., Yarmoluk S.M. (2005). Fluorescent homodimer styrylcyanines: Synthesis and spectral-luminescent studies in nucleic acids and protein complexes. Dyes Pigments.

[126] Yarmoluk S., Kovalska V., Losytskyy M. (2008). Symmetric cyanine dyes for detecting nucleic acids. Biotech. Histochem..

[127] Didenko V.V. (2001). DNA probes using fluorescence resonance energy transfer (FRET): Designs and applications. BioTechniques.

[128] Foister S. (2003). Shape Selective Recognition of the DNA Minor Groove by Hairpin Polyamides. Thesis for the Degree of Doctor of Philosophy.

[129] Stadler A.L., Delos Santos J.O., Stensrud E.S., Dembska A., Silva G.L., Liu S.P., Shank N.I., Kunttas-Tatli E., Sobers C.J., Gramlich P.M.E. (2011). Fluorescent DNA nanotags featuring covalently attached intercalating dyes: Synthesis, antibody conjugation, and intracellular imaging. Bioconjug. Chem..

[130] Kim M.Y., Kim J., Hah S.S. (2012). Poly(A)-targeting molecular beacons: Fluorescence resonance energy transfer-based *in vitro* quantitation and time-dependent imaging in live cells. Anal. Biochem..

[131] Duhamel J. (2012). New insights in the study of pyrene excimer fluorescence to characterize macromolecules and their supramolecular assemblies in solution. Langmuir.

[132] Krasheninina O.A., Novopashina D.S., Venyaminova A.G. (2011). Oligo(2'-*O*-methylribonucleotides) containing insertions of 2'-bispyrenylmethylphosphoro-diamidate nucleoside derivatives as prospective fluorescent probes for RNA detection. Russ. J. Bioorg. Chem..

[133] Novopashina D.S., Meschaninova M.I., Kholodar S.A., Lomzov A.A., Venyaminova A.G. (2008). New eximer-based tandem systems for SNP detection. Nucleic Acids Symp. Ser..

[134] Kholodar S.A., Novopashina D.S., Meschaninova M.I., Lomzov A.A., Venyaminova A.G. (2009). Multipyrene tandem probes for detection of C677T polymorphism in MTHFR gene. Nucleic Acids Symp. Ser..

[135] Kolpashchikov D.M. (2010). Binary probes for nucleic acid analysis. Chem. Rev..

[136] Novikova I.V., Afonin K.A., Leontis N.B., Serra P.A. (2010). New Ideas for *in Vivo* Detection of RNA. Biosensors.

[137] Guo J., Ju J., Turro N.J. (2012). Fluorescent hybridization probes for nucleic acid detection. Anal. Bioanal. Chem..

[138] Teo Y.N., Wilson J.N., Kool E.T. (2009). Polyfluorophores on a DNA backbone: A multicolor set of labels excited at one wavelength. J. Am. Chem. Soc..

[139] Wang S., Guo J., Ono T., Kool E.T. (2012). DNA polyfluorophores for real-time multicolor tracking of dynamic biological systems. Angew. Chem. Int. Ed..

[140] Guo J., Wang S., Dai N., Teo Y.N., Kool E.T. (2011). Multispectral labeling of antibodies with polyfluorophores on a DNA backbone and application in cellular imaging. Proc. Nat. Acad. Sci. USA.

[141] Teo Y.N., Kool E.T. (2012). DNA-multichromophore systems. Chem. Rev..

[142] Ponting C.P., Oliver P.L., Reik W. (2009). Evolution and functions of long noncoding RNAs. Cell.

[143] Kruger K., Grabowski P.J., Zaug A.J., Sands J., Gottschling D.E., Cech T.R. (1982). Self-splicing RNA: Autoexcision and autocyclization of the ribosomal RNA intervening sequence of tetrahymena. Cell.

[144] Guerrier-Takada C., Gardiner K., Marsh T., Pace N., Altman S. (1983). The RNA moiety of ribonuclease P is the catalytic subunit of the enzyme. Cell.

[145] Green P.J., Pines O., Inouye M. (1986). The role of antisense RNA in gene regulation. Annu. Rev. Biochem..

[146] The FANTOM Consortium (2005). The transcriptional landscape of the mammalian genome. Science.

[147] Bushati N., Cohen S.M. (2007). MicroRNA functions. Ann. Rev. Cell Dev. Biol..

[148] Wang Y., Stricker H.M., Gou D.M., Liu L. (2007). MicroRNA: Past and present. Front. Biosci..

[149] Bouzinba-Segard H., Guais A., Francastel C. (2006). Accumulation of small murine minor satellite transcripts leads to impaired centromeric architecture and function. Proc. Nat. Acad. Sci. USA.

[150] Thorsen M., Hansen H., Venturi M., Holmberg S., Thon G. (2012). Mediator regulates non-coding RNA transcription at fission yeast centromeres. Epigenetics Chromatin.

[151] Carlsten J.O., Szilagyi Z., Liu B., Lopez M.D., Szászi E., Djupedal I., Nyström T., Ekwall K., Gustafsson C.M., Zhu X. (2012). Mediator promotes CENP-a incorporation at fission yeast centromeres. Mol. Cell. Biol..

[152] Enukashvily N.I., Malashicheva A.B., Waisertreiger I.S.R. (2009). Satellite DNA spatial localization and transcriptional activity in mouse embryonic E-14 and IOUD2 stem cells. Cytogenet. Genome Res..

[153] Chan F.L., Wong L.H. (2012). Transcription in the maintenance of centromere chromatin identity. Nucleic Acids Res..

[154] Chan F.L., Marshall O.J., Saffery R., Won Kim B., Earle E., Choo K.H.A., Wong L.H. (2012). Active transcription and essential role of RNA polymerase II at the centromere during mitosis. Proc. Nat. Acad. Sci. USA.

[155] Gent J.I., Dawe R.K. (2012). RNA as a structural and regulatory component of the centromere. Annu. Rev. Genet..

[156] Hall L.E., Mitchell S.E., O’Neill R.J. (2012). Pericentric and centromeric transcription: A perfect balance required. Chromosome Res..

[157] Dictenberg J. (2012). Genetic encoding of fluorescent RNA ensures a bright future for visualizing nucleic acid dynamics. Trends Biotechnol..

[158] Tyagi S. (2009). Imaging intracellular RNA distribution and dynamics in living cells. Nat. Methods.

[159] Bertrand E., Chartrand P., Schaefer M., Shenoy S.M., Singer R.H., Long R.M. (1998). Localization of ASH1 mRNA particles in living yeast. Mol. Cell.

[160] Beach D.L., Salmon E.D., Bloom K. (1999). Localization and anchoring of mRNA in budding yeast. Curr. Biol..

[161] Haim L., Zipor G., Aronov S., Gerst J.E. (2007). A genomic integration method to visualize localization of endogenous mRNAs in living yeast. Nat. Methods.

[162] Bao G., Rhee W.J., Tsourkas A. (2009). Fluorescent probes for live-cell RNA detection. Ann. Rev. Biomed. Eng..

[163] Dirks R.W., Molenaar C., Tanke H.J. (2003). Visualizing RNA molecules inside the nucleus of living cells. Methods.

[164] Bao G., Santangelo P., Nitin N., Rhee W.J., Fuchs H., Grätzel M., Krug H., Schmid G., Vogel V., Waser R. (2010). Nanostructured Probes for *in Vivo* Gene Detection. Nanotechnology.

[165] Ranasinghe R.T., Brown L.J., Brown T. (2001). Linear fluorescent oligonucleotide probes with an acridine quencher generate a signal upon hybridisation. Chem. Commun..

[166] Waki R., Yamayoshi A., Kobori A., Murakami A. (2011). Development of a system to sensitively and specifically visualize c-fos mRNA in living cells using bispyrene-modified RNA probes. Chem. Commun..

[167] Menacher F., Rubner M., Berndl S., Wagenknecht H.-A. (2008). Thiazole orange and Cy3: Improvement of fluorescent DNA probes with use of short range electron transfer. J. Org. Chem..

[168] Bethge L., Singh I., Seitz O. (2010). Designed thiazole orange nucleotides for the synthesis of single labelled oligonucleotides that fluoresce upon matched hybridization. Org. Biomol. Chem..

[169] Holzhauser C., Wagenknecht H.A. (2012). “DNA traffic lights”: Concept of wavelength-shifting DNA probes and application in an aptasensor. ChemBioChem.

[170] Holzhauser C., Berndl S., Menacher F., Breunig M., Göpferich A., Wagenknecht H.-A. (2010). Synthesis and optical properties of cyanine dyes as fluorescent DNA base substitutions for live cell imaging. Eur. J. Org. Chem..

[171] Holzhauser C., Liebl R., Goepferich A., Wagenknecht H.-A., Breunig M. (2013). RNA “Traffic lights”: An analytical tool to monitor siRNA integrity. ACS Chem. Biol..

[172] Kashida H., Takatsu T., Sekiguchi K., Asanuma H. (2010). An efficient fluorescence resonance energy transfer (FRET) between pyrene and perylene assembled in a DNA duplex and its potential for discriminating single-base changes. Chem. Eur. J..

[173] Kato T., Kashida H., Kishida H., Yada H., Okamoto H., Asanuma H. (2012). Development of a robust model system of FRET using base surrogates tethering fluorophores for strict control of their position and orientation within DNA duplex. J. Am. Chem. Soc..

[174] Ikeda S., Okamoto A. (2008). Hybridization-sensitive on–off DNA probe: Application of the exciton coupling effect to effective fluorescence quenching. Chem. Asian J..

[175] Kubota T., Ikeda S., Yanagisawa H., Yuki M., Okamoto A. (2009). Hybridization-sensitive fluorescent probe for long-term monitoring of intracellular RNA. Bioconjug. Chem..

[176] Köhler O., Seitz O. (2003). Thiazole orange as fluorescent universal base in peptide nucleic acids. Chem. Commun..

[177] Köhler O., Jarikote D.V., Seitz O. (2005). Forced intercalation probes (FIT probes): Thiazole orange as a fluorescent base in peptide nucleic acids for homogeneous single-nucleotide-polymorphism detection. ChemBioChem.

[178] Kummer S., Knoll A., Socher E., Bethge L., Herrmann A., Seitz O. (2011). Fluorescence imaging of influenza H1N1 mRNA in living infected cells using single-chromophore FIT-PNA. Angew. Chem. Int. Ed..

[179] Kummer S., Knoll A., Socher E., Bethge L., Herrmann A., Seitz O. (2012). PNA FIT-probes for the dual color imaging of two viral mRNA targets in influenza H1N1 infected live cells. Bioconjug. Chem..

[180] Tyagi S., Kramer F.R. (1996). Molecular beacons: Probes that fluoresce upon hybridization. Nat. Biotechnol..

[181] Tan W., Wang K., Drake T.J. (2004). Molecular beacons. Curr. Opin. Chem. Biol..

[182] Huang K., Martí A. (2012). Recent trends in molecular beacon design and applications. Anal. Bioanal. Chem..

[183] Tsourkas A., Behlke M.A., Bao G. (2002). Hybridization of 2'-O-methyl and 2'-deoxy molecular beacons to RNA and DNA targets. Nucleic Acids Res..

[184] Tsourkas A., Behlke M.A., Bao G. (2002). Structure–function relationships of shared-stem and conventional molecular beacons. Nucleic Acids Res..

[185] Tsourkas A., Behlke M.A., Rose S.D., Bao G. (2003). Hybridization kinetics and thermodynamics of molecular beacons. Nucleic Acids Res..

[186] Rhee W.J., Bao G. (2010). Slow non-specific accumulation of 2'-deoxy and 2'-O-methyl oligonucleotide probes at mitochondria in live cells. Nucleic Acids Res..

[187] Rhee W.J., Bao G. (2009). Simultaneous detection of mRNA and protein stem cell markers in live cells. BMC Biotechnol..

[188] Chen A.K., Davydenko O., Behlke M.A., Tsourkas A. (2010). Ratiometric bimolecular beacons for the sensitive detection of RNA in single living cells. Nucleic Acids Res..

[189] Molenaar C., Marras S.A., Slats J.C.M., Truffert J.-C., Lemaïtre M., Raap A.K., Dirks R.W., Tanke H.J. (2001). Linear 2'-O-Methyl RNA probes for the visualization of RNA in living cells. Nucleic Acids Res..

[190] Liu L., Tang Z., Wang K., Tan W., Li J., Guo Q., Meng X., Ma C. (2005). Using molecular beacon to monitor activity of *E. coli* DNA ligase. Analyst.

[191] Tang Z., Wang K., Tan W., Li J., Liu L., Guo Q., Meng X., Ma C., Huang S. (2003). Real-time monitoring of nucleic acid ligation in homogenous solutions using molecular beacons. Nucleic Acids Res..

[192] Ma C., Tang Z., Wang K., Tan W., Yang X., Li W., Li Z., Lv X. (2007). Real-time monitoring of restriction endonuclease activity using molecular beacon. Anal. Biochem..

[193] Ma C., Tang Z., Wang K., Tan W., Yang X., Li W., Li Z., Li H., Lv X. (2007). Real-time monitoring of nucleic acid dephosphorylation by using molecular beacons. ChemBioChem.

[194] Zhang C., Su X., Liang Y., Zhu X., Song C., Zhao M. (2011). A transformer of molecular beacon for sensitive and real-time detection of phosphatases with effective inhibition of the false positive signals. Biosens. Bioelectron..

[195] Ma C., Tang Z., Wang K., Tan W., Li J., Li W., Li Z., Yang X., Li H., Liu L. (2006). Real-time monitoring of DNA polymerase activity using molecular beacon. Anal. Biochem..

[196] Li J., Yan H., Wang K., Tan W., Zhou X. (2007). Hairpin fluorescence DNA probe for real-time monitoring of DNA methylation. Anal. Chem..

[197] Tang Z., Wang K., Tan W., Ma C., Li J., Liu L., Guo Q., Meng X. (2005). Real-time investigation of nucleic acids phosphorylation process using molecular beacons. Nucleic Acids Res..

[198] Ma C., Yang X., Wang K., Tang Z., Li W., Tan W., Lv X. (2008). A novel kinase-based ATP assay using molecular beacon. Anal. Biochem..

[199] Fujimoto K., Shimizu H., Inouye M. (2004). Unambiguous detection of target DNAs by excimer-monomer switching molecular beacons. J. Org. Chem..

[200] Huang J., Wu Y., Chen Y., Zhu Z., Yang X., Yang C.J., Wang K., Tan W. (2011). Pyrene-excimer probes based on the hybridization chain reaction for the detection of nucleic acids in complex biological fluids. Angew. Chem. Int. Ed..

[201] Yamana K., Ohshita Y., Fukunaga Y., Nakamura M., Maruyama A. (2008). Bis-pyrene-labeled molecular beacon: A monomer–excimer switching probe for the detection of DNA base alteration. Bioorg. Med. Chem..

[202] Biner S.M., Häner R. (2011). A two-color, self-controlled molecular beacon. ChemBioChem.

[203] Xiang D., Zhang C., Chen L., Ji X., He Z. (2012). Tricolour fluorescence detection of sequence-specific DNA with a new molecular beacon and a nucleic acid dye TOTO-3. Analyst.

[204] Yang C.J., Martinez K., Lin H., Tan W. (2006). Hybrid molecular probe for nucleic acid analysis in biological samples. J. Am. Chem. Soc..

[205] Zhu G., Zhang S., Song E., Zheng J., Hu R., Fang X., Tan W. (2013). Building fluorescent DNA nanodevices on target living cell surfaces. Angew. Chem. Int. Ed..

[206] Varghese R., Wagenknecht H.-A. (2010). Red-white-blue emission switching molecular beacons: Ratiometric multicolour DNA hybridization probes. Org. Biomol. Chem..

[207] Urano Y., Asanuma D., Hama Y., Koyama Y., Barrett T., Kamiya M., Nagano T., Watanabe T., Hasegawa A., Choyke P.L. (2009). Selective molecular imaging of viable cancer cells with pH-activatable fluorescence probes. Nat. Med..

[208] Kashida H., Yamaguchi K., Hara Y., Asanuma H. (2012). Quencher-free molecular beacon tethering 7-hydroxycoumarin detects targets through protonation/deprotonation. Bioorg. Med. Chem..

[209] Su X., Zhang C., Zhao M. (2011). Discrimination of the false-positive signals of molecular beacons by combination of heat inactivation and using single walled carbon nanotubes. Biosens. Bioelectron..

[210] Petersen K., Vogel U., Rockenbauer E., Vang Nielsen K., Kølvraa S., Bolund L., Nexø B. (2004). Short PNA molecular beacons for real-time PCR allelic discrimination of single nucleotide polymorphisms. Mol. Cell. Probes.

[211] Kim Y., Yang C.J., Tan W. (2007). Superior structure stability and selectivity of hairpin nucleic acid probes with an L-DNA stem. Nucleic Acids Res..

[212] Yang C.J., Wang L., Wu Y., Kim Y., Medley C.D., Lin H., Tan W. (2007). Synthesis and investigation of deoxyribonucleic acid/locked nucleic acid chimeric molecular beacons. Nucleic Acids Res..

[213] Wang L., Yang C.J., Medley C.D., Benner S.A., Tan W. (2005). Locked nucleic acid molecular beacons. J. Am. Chem. Soc..

[214] Martinez K., Estevez M.C., Wu Y., Phillips J.A., Medley C.D., Tan W. (2009). Locked nucleic acid based beacons for surface interaction studies and biosensor development. Anal. Chem..

[215] Chen A.K., Behlke M.A., Tsourkas A. (2009). Sub-cellular trafficking and functionality of 2'-O-methyl and 2'-O-methyl-phosphorothioate molecular beacons. Nucleic Acids Res..

[216] Qiu L., Wu C., You M., Han D., Chen T., Zhu G., Jiang J., Yu R., Tan W. (2013). A targeted, self-delivered, and photocontrolled molecular meacon for mRNA detection in living cells. J. Am. Chem. Soc..

[217] Okabe K., Harada Y., Zhang J., Tadakuma H., Tani T., Funatsu T. (2011). Real time monitoring of endogenous cytoplasmic mRNA using linear antisense 2'-O-methyl RNA probes in living cells. Nucleic Acids Res..

[218] Martí A.A., Li X., Jockusch S., Li Z., Raveendra B., Kalachikov S., Russo J.J., Morozova I., Puthanveettil S.V., Ju J. (2006). Pyrene binary probes for unambiguous detection of mRNA using time-resolved fluorescence spectroscopy. Nucleic Acids Res..

[219] Silverman A.P., Abe H., Kool E.T., Marx A., Seitz O. (2008). Quenched Autoligation (QUAL) Probes. Molecular Beacons: Signalling Nucleic Acid Probes, Methods, and Protocols.

[220] Sando S., Kool E.T. (2002). Imaging of RNA in bacteria with self-ligating quenched probes. J. Am. Chem. Soc..

[221] Abe H., Kool E.T. (2004). Destabilizing universal linkers for signal amplification in self-ligating probes for RNA. J. Am. Chem. Soc..

[222] Abe H., Kool E.T. (2006). Flow cytometric detection of specific RNAs in native human cells with quenched autoligating FRET probes. Proc. Nat. Acad. Sci. USA.

[223] Silverman A.P., Baron E.J., Kool E.T. (2006). RNA-templated chemistry in cells: Discrimination of Escherichia, Shigella and Salmonella bacterial strains with a new two-color FRET strategy. ChemBioChem.

[224] Silverman A.P., Kool E.T. (2005). Quenched autoligation probes allow discrimination of live bacterial species by single nucleotide differences in rRNA. Nucleic Acids Res..

[225] Miller G.P., Silverman A.P., Kool E.T. (2008). New, stronger nucleophiles for nucleic acid-templated chemistry: Synthesis and application in fluorescence detection of cellular RNA. Bioorg. Med. Chem..

[226] Franzini R.M., Kool E.T. (2009). Efficient nucleic acid detection by templated reductive quencher release. J. Am. Chem. Soc..

[227] Kleinbaum D.J., Kool E.T. (2010). Sandwich probes: Two simultaneous reactions for templated nucleic acid detection. Chem. Commun..

[228] Franzini R.M., Kool E.T. (2011). Two successive reactions on a DNA template: A strategy for improving background fluorescence and specificity in nucleic acid detection. Chem. Eur. J..

[229] Franzini R.M., Kool E.T. (2011). Improved templated fluorogenic probes enhance the analysis of closely related pathogenic bacteria by microscopy and flow cytometry. Bioconjug. Chem..

[230] Röthlingshöfer M., Gorska K., Winssinger N. (2011). Nucleic acid-templated energy transfer leading to a photorelease reaction and its application to a system displaying a nonlinear response. J. Am. Chem. Soc..

[231] Röthlingshöfer M., Gorska K., Winssinger N. (2011). Nucleic acid templated uncaging of fluorophores using Ru-catalyzed photoreduction with visible light. Org. Lett..

[232] Sadhu K.K., Winssinger N. (2013). Detection of miRNA in live cells by using templated Ru(II)-catalyzed unmasking of a fluorophore. Chem. Eur. J..

[233] Grossmann T.N., Seitz O. (2006). DNA-catalyzed transfer of a reporter group. J. Am. Chem. Soc..

[234] Grossmann T.N., Roglin L., Seitz O. (2008). Target-catalyzed transfer reactions for the amplified detection of RNA. Angew. Chem. Int. Ed..

[235] Chen X.H., Roloff A., Seitz O. (2012). Consecutive signal amplification for DNA detection based on *de novo* fluorophore synthesis and host-guest chemistry. Angew. Chem. Int. Ed..

[236] Syed M.A., Pervaiz S. (2010). Advances in aptamers. Oligonucleotides.

[237] Ni X., Castanares M., Mukherjee A., Lupold S.E. (2011). Nucleic acid aptamers: Clinical applications and promising new horizons. Curr. Med. Chem..

[238] Mascini M., Palchetti I., Tombelli S. (2012). Nucleic acid and peptide aptamers: Fundamentals and bioanalytical aspects. Angew. Chem. Int. Ed..

[239] Vendrell M., Zhai D., Er J.C., Chang Y.-T. (2012). Combinatorial strategies in fluorescent probe development. Chem. Rev..

[240] Kolpashchikov D.M. (2005). Binary malachite green aptamer for fluorescent detection of nucleic acids. J. Am. Chem. Soc..

[241] Sando S., Narita A., Aoyama Y. (2007). Light-up hoechst–DNA aptamer pair: Generation of an aptamer-selective fluorophore from a conventional DNA-staining dye. ChemBioChem.

[242] Eydeler K., Magbanua E., Werner A., Ziegelmüller P., Hahn U. (2009). Fluorophore binding aptamers as a tool for RNA visualization. Biophys. J..

[243] Paige J.S., Wu K.Y., Jaffrey S.R. (2011). RNA Mimics of green fluorescent protein. Science.

[244] Sassolas A., Blum L.J., Leca-Bouvier B.D. (2011). Homogeneous assays using aptamers. Analyst.

[245] Astakhova I.V., Korshun V.A., Jahn K., Kjems J., Wengel J. (2008). Perylene attached to 2'-amino-LNA: Synthesis, incorporation into oligonucleotides, and remarkable fluorescence properties *in vitro* and in cell culture. Bioconjug. Chem..

[246] Cummins L.L., Owens S.R., Risen L.M., Lesnik E.A., Freier S.M., McGee D., Guinosso C.J., Cook P.D. (1995). Characterization of fully 2'-modified oligoribonucleotide hetero-and homoduplex hybridization and nuclease sensitivity. Nucleic Acids Res..

[247] Novopashina D., Sinyakov A., Ryabinin V., Venyaminova A., Boutorine A. (2003). Conjugates of oligo(2'-O-methylribonucleotides) with minor groove binders as new sequence-specific agents recognizing both grooves of double-stranded DNA. Nucleosides Nucleotides Nucleic Acids.

[248] Novopashina D.S., Sinyakov A.N., Ryabinin V.A., Venyaminova A.G., Halby L., Sun J.S., Boutorine A.S. (2005). Sequence-specific conjugates of oligo(2'-O-methylribonucleotides) and hairpin oligocarboxamide minor-groove binders: Design, synthesis, and binding studies with double-stranded DNA. Chem. Biodivers..

[249] Boutorine A.S., Venyaminova A.G., Repkova M.N., Sergueyeva Z.A., Pyshnyi D.V. (1994). Effect of derivatization of ribophosphate backbone and terminal ribophosphate groups in oligoribonucleotides an their stability and interaction with eukaryotic cells. Biochimie.

[250] Novopashina D., Kuznetsova M., Venyaminova A. (2001). 2'-O-modified oligoribonucleotides with terminal 3'-3'-internucleotide linkage and their derivatives. Nucleosides Nucleotides Nucleic Acids.

[251] Novopashina D.S., Totskaya O.S., Lomzov A.A., Venyaminova A.G. (2005). 3'-modified oligo(2'-O-methylribonucleotides) as improved probes for hybridization with RNA. Nucleosides Nucleotides Nucleic Acids.

[252] Majlessi M., Nelson N.C., Becker M.M. (1998). Advantages of 2'-O-methyl oligoribonucleotide probes for detecting RNA targets. Nucleic Acids Res..

[253] Bratu D.P., Cha B.J., Mhlanga M.M., Kramer F.R., Tyagi S. (2003). Visualizing the distribution and transport of mRNAs in living cells. Proc. Nat. Acad. Sci. USA.

[254] Malik R., Roy I. (2011). Making sense of therapeutics using antisense technology. Expert Opin. Drug Discov..

[255] Juliano R.L., Ming X., Nakagawa O. (2011). Cellular uptake and intracellular trafficking of antisense and siRNA oligonucleotides. Bioconjug. Chem..

[256] Lochmann D., Jauk E., Zimmer A. (2004). Drug delivery of oligonucleotides by peptides. Eur. J. Pharm. Biopharm..

[257] Elsabahy M., Nazarali A., Foldvari M. (2011). Non-viral nucleic acid delivery: Key challenges and future directions. Curr. Drug Deliv..

[258] Lacerda L., Bianco A., Prato M., Kostarelos K. (2008). Carbon nanotube cell translocation and delivery of nucleic acids *in vitro* and *in vivo*. J. Mater. Chem..

[259] Campidelli S., Ballesteros B., Filoramo A., Díaz D.D., de la Torre G., Torres T., Rahman G.M.A., Ehli C., Kiessling D., Werner F. (2008). Facile decoration of functionalized single-wall carbon nanotubes with phthalocyanines via “click chemistry”. J. Am. Chem. Soc..

[260] Brunetti F.G., Herrero M.A., Munoz J.D., Diaz-Ortiz A., Alfonsi J., Meneghetti M., Prato M., Vazquez E. (2008). Microwave-induced multiple functionalization of carbon nanotubes. J. Am. Chem. Soc..

[261] Prato M., Kostarelos K., Bianco A. (2008). Functionalized carbon nanotubes in drug design and discovery. Acc. Chem. Res..

[262] Apartsin E.K., Novopashina D.S., Nastaushev Y.V., Venyaminova A.G. (2012). Fluorescently labeled single-walled carbon nanotubes and their hybrids with oligonucleotides. Nanotechnol. Russ..

[263] Apartsin E.K., Buyanova M.Y., Novopashina D.S., Ryabchikova E.I., Venyaminova A.G., Fesenko O., Yatsenko L., Brodin M. (2013). Non-Covalent Immobilization of Oligonucleotides on Single-Walled Carbon Nanotubes. Nanomaterials Imaging Techniques, Surface Studies, and Applications.

